# Biological Evaluation of Natural and Synthesized Homovanillic Acid Esters as Inhibitors of Intestinal Fatty Acid Uptake in Differentiated Caco-2 Cells

**DOI:** 10.3390/molecules24193599

**Published:** 2019-10-07

**Authors:** Barbara Lieder, Joachim Hans, Fabia Hentschel, Katrin Geissler, Jakob Ley

**Affiliations:** 1 Symrise AG, Muehlenfeldstrasse 1, 53479 Holzminden, Germany; 2 Department of Physiological Chemistry, University of Vienna, CDL for Taste Research, Althanstrasse 14, Vienna 1090, Austria

**Keywords:** fatty acid uptake, capsiate, homovanillic acid esters, capsaicin, Caco-2 cells, obesity

## Abstract

With raising prevalence of obesity, the regulation of human body fat is increasingly relevant. The modulation of fatty acid uptake by enterocytes represents a promising target for body weight maintenance. Recent results demonstrated that the trigeminal active compounds capsaicin, nonivamide, and *trans*-pellitorine dose-dependently reduce fatty acid uptake in differentiated Caco-2 cells as a model for the intestinal barrier. However, non-pungent alternatives have not been investigated and structural determinants for the modulation of intestinal fatty acid uptake have not been identified so far. Thus, based on the previous results, we synthesized 23 homovanillic acid esters in addition to the naturally occurring capsiate and screened them for their potential to reduce intestinal fatty acid uptake using the fluorescent fatty acid analog Bodipy-C12 in differentiated Caco‑2 cells as an enterocyte model. Whereas pre-incubation with 100 µM capsiate did not change fatty acid uptake by Caco-2 enterocytes, a maximum inhibition of −47% was reached using 100 µM 1‑methylpentyl-2-(4-hydroxy-3-methoxy-phenyl)acetate. Structural analysis of the 24 structural analogues tested in the present study revealed that a branched fatty acid side chain, independent of the chain length, is one of the most important structural motifs associated with inhibition of fatty acid uptake in Caco-2 enterocytes. The results of the present study may serve as an important basis for designing potent dietary inhibitors of fatty acid uptake.

## 1. Introduction

With a raising prevalence of obesity worldwide, the regulation of human body fat is of increasing interest. One accepted measure for weight loss is blocking dietary fat absorption [1]. Most of the common drugs are based on inhibitors for pancreatic lipase activity, thus decreasing digestion of triglycerides, and resulting in a reduced fatty acid uptake. However, inhibition of lipase activity is associated with major side effects such as a reduced absorption of lipid-soluble vitamins and digestive disorders [2]. Thus, current research focuses on compounds specifically targeting mechanisms of fatty acid uptake, which allows maintaining normal digestion of triglycerides and absorption of lipid-soluble nutrients [3]. Fatty acid uptake is accomplished by enterocytes in the small intestine at the brush border membrane [4]. The exact molecular mechanisms underlying intestinal fatty acid uptake are not completely understood yet, but diffusion seems to coexist with protein-mediated mechanisms such as fatty acid transporters. Current research suggests fatty acid transport protein (FATP) 4, fatty acid binding proteins, as well as the fatty acid translocase (CD36) for a facilitated membrane transport to be the most relevant protein-mediated mechanisms in enterocytes [4]. A widely studied model for investigating nutrient uptake by enterocytes of the small intestine are differentiated Caco-2 cells, as they exhibit many characteristics of mature villus epithelial cells upon differentiation [5]. For example, differentiated Caco-2 cells express tight junctions, microvilli, and brush border membrane enzymes such as peptidases and esterases [6,7]. Moreover, since differentiated Caco-2 cells express FATP2, FATP4, and CD36, as well as PPARα and PPARγ [8,9], they have been used as a model for studying mechanisms of fatty acid uptake in previous studies [8,9,10,11,12].

**Figure 1:**
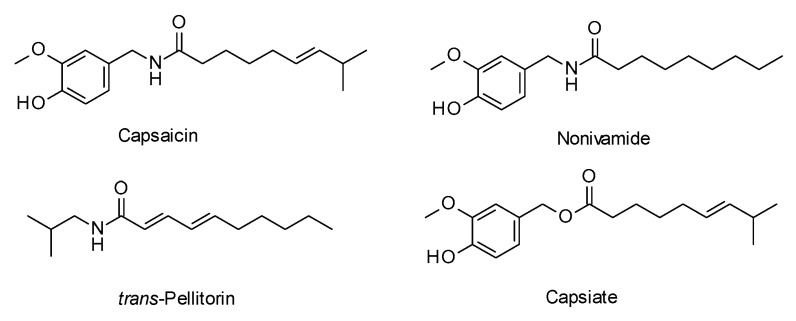
Structures of capsaicin, nonivamide, *trans*-pellitorine, and capsiate.

Previous studies aiming to identify fatty acid uptake reducing food ingredients demonstrated that the alkamides capsaicin, nonivamide and *trans*-pellitorine inhibited fatty acid uptake in differentiated Caco-2 cells as a model for intestinal enterocytes, with different potency [12]. The most pronounced effect was detected for capsaicin, followed by nonivamide and *trans*-pellitorine (Figure 1). For the tested alkamides, an impact on passive diffusion and membrane integrity was excluded and a direct interaction with the fatty acid transport proteins hypothesized. However, capsaicin, nonivamide, and trans-pellitorine are well-known for their strong trigeminal responses when orally ingested, which is limiting their dietary intake [10,13]. A recent molecular docking study indicated a lower binding efficiency of capsiate in comparison to capsaicin to the transient receptor potential cation channel V1 (TRPV1) channel [14], the receptor responsible for the pungent sensation of capsaicinoids [12]. However, the previous performed study on fatty acid uptake inhibition by alkamides suggested that the inhibitory potential is independent of activation of the TRPV1 ion channel. This fact is giving rise to the question whether also non-pungent *O*-analogs of capsaicin, such as capsiate (Figure 1), have a similar potential to inhibit intestinal fatty acid uptake. Although activation of TRPV1 has been suggested to be crucial for the anti-obesity effects of capsaicin [15], the non-pungent capsiate has been shown to have an impact on lipid metabolism as well: Mice fed a high or low fat diet supplemented with 5% capsionoids for 12 weeks showed an increased expression of CD36, a protein that facilitates fatty acid uptake, in adipose tissue and the liver [16]. 

Different homovanillic acid esters as structural relatives of capsionoids have been recently described to exhibit mild chemesthetic effects [17,18], however, bioactivity regarding their impact on lipid metabolism has not been investigated so far. Moreover, information on structural determinants which are beneficial to reduce fatty acid uptake in Caco-2 enterocytes is very limited. Thus, in the present study, we aimed to analyze which structural motifs of the fatty acid side chain determine a compound’s fatty acid uptake inhibitory potential. For this purpose, a total of 23 homovanillic acid esters was synthesized and evaluated for their potential as intestinal fatty acid uptake inhibitor using differentiated Caco-2 cells as a model for the intestinal barrier.

## 2. Results and Discussion

**Figure 2:**
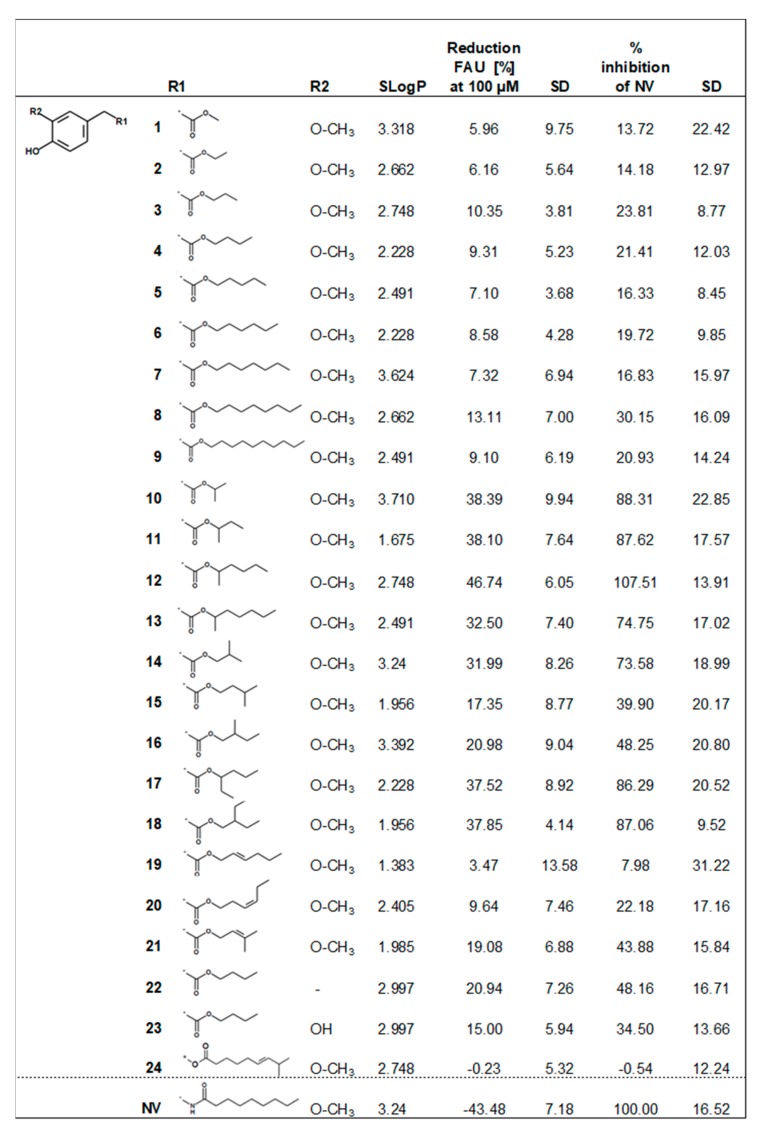
Overview of the tested naturally occurring and synthesized homovanillic acid esters, their SLogP values, and their potency to reduce fatty acid uptake (FAU) at 100 µM in relation to non-treated control cells (0% inhibition) or as % of the positive control nonivamide (NV, −43% inhibition of control level). Displayed is the mean of three independent experiments with two technical replicates per measurement for each compound.

A total of 23 homovanillic acid esters was synthesized in addition to capsiate (**24**) in order to investigate which structural motifs are advantageous to inhibit intestinal fatty acid uptake. The previously reported active and structurally related compound nonivamide was used as a positive control. Figure 2 provides an overview of the test compounds used in the present study.

### 2.1. Synthesis of Homovanillate Esters

**Figure 3:**
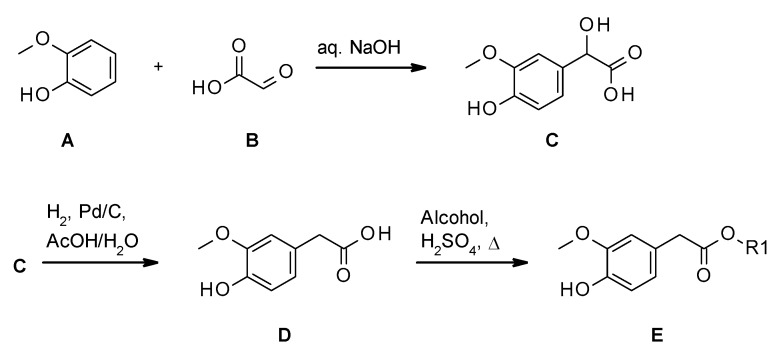
Schematic overview of the general synthesis to homovanillic esters **D**.

The synthesis of 2-(4-hydroxy-3-methoxyphenyl) acetic acid (homovanillic acid, **D**) involved two steps (see Figure 3), following the procedure described by Umumura et al. [19], with slight modifications (Figure 2). First, mandelic acid **C** was synthesized from guaiacol **A** and glyoxylic acid **B** in the presence of NaOH solution. The product was isolated by extracting the product with ethyl acetate and followed by distillation. Finally, the product was isolated in the mixture of *n*-hexane and ethyl acetate. The typical isolated yield of the reaction was in the range of 50%–54%. Acid **D** was synthesized from compound **C** using 5% Palladium on carbon (50.0% wet and 2.5 *w/w* loading), hydrogen gas, acetic acid, and water. The product was isolated by distillation and followed by crystallization. The typical isolated yield of the reaction was in the range of 74%. Esterification to compound class **D** was performed in an excess of the corresponding alcohol or in a Dean-Stark apparatus. The typical isolated yields after purification by distillation or column chromatography (silica, *n*-hexane/ethyl acetate) were >70%.

### 2.2. Biological Evaluation

**Figure 4:**
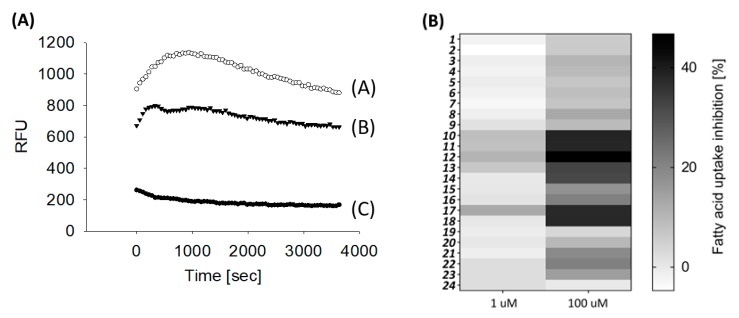
(**A**) Typical fatty acid uptake measurement in differentiated Caco-2 cells showing the respective time-intensity plots for control-treated cells (white circles, A), compound treated cells (black triangles, B), or cell free measurements (black dots, C). (**B**) Overview of the reduction of fatty acid uptake induced by the tested compounds shown as area under the curves (ΔAUC) in %, (control = 0). Differentiated Caco-2 cells were pre-treated with the 24 homovanillic acid esters at one (left side) or 100 µM (right side) for 30 min and the AUC calculated from the respective time-intensity plots as shown exemplarily at Figure 4A. Displayed is the mean of three independent measurements with two technical replicates for each measurement per compound and concentration.

The synthesized compounds were tested regarding their potential to modulate fatty acid uptake in differentiated Caco-2 cells at one and 100 µM and the area under the curve was calculated from the respective time-intensity plots (Figure 4A). 

Two-Way ANOVA with Holm-Sidak Post hoc test revealed a concentration-dependent effect for all tested homovanillic acid esters except for the compound (**24**), capsiate, which did not influence fatty acid uptake in the tested concentration range. Figure 4B provides an overview of the fatty acid uptake inhibiting potential of all test compounds at one and 100 µM and displays the concentration-dependency as a heat-map. The following analyses to identify important structural motifs to reduce fatty acid uptake were carried out with data obtained after treatment with the more potent test concentration (100 µM), assuming higher or lower activities based on statistical significant differences in fatty acid uptake inhibition, which were assessed by One-Way ANOVA followed by Holm-Sidak post hoc test. 

First, the impact of the chain length of the fatty acid side chain was analyzed using a total of nine compounds with a saturated, non-branched fatty acid side chain with a chain length ranging from C1 (**1**) to C10 (**9**) (Figure 2). A chain length >10 will lead to low water solubility and reduced bioavailability [20] and was therefore not tested. The chain length of the carbon side chain of the test compounds did not correlate with the potency to inhibit fatty acid uptake (Pearson correlation, R = 0.231, *p* > 0.05), leading to the conclusion that the chain length is not the most important structural determinant. However, from the non-branched test compounds, only pre-treatment with test compound (**8**) led to a reduced fatty acid uptake versus control treated cells by 13%. With eight C-atoms, the chain length of (**8**) is in the range of the optimal lipophilicity and water solubility regarding bioavailability and penetration of cell membranes [20]. 

The impact of branching in the alkyl chain was evaluated with another set of test compounds (**10**–**18**). Branching increases molar volume, which may also lead to a reduction in lipophilicity, and adds new stereogenic centers and might therefore have larger impact on the bioactivity of test compounds [20]. Methyl-branching at C1 of the ester function was introduced to four compounds with a carbon chain with three to six carbons. All of the tested C1-methyl-branched molecules showed a significantly increased inhibitory potential compared to their unbranched direct structural analogues (Figure 2). Moreover, compound (**10**) with a chain length of C2 (−38%) was more potent than derivative (**15**) with a chain length of C4 (−17%) (*p* < 0.001) and similar effective as an isobutyl-residue (−32%) in compound (**14**), 1-methylhexyl-residue (−33%) in ester (**13**) or 1‑methylpropyl-residue (−38%) in derivative (**11**) (*p* < 0.05). However, the strongest effect was detected for compound (**12**), with a 1-methylpentyl-residue. It has to be noticed that with a reduction of −47%, the results obtained for the ester (**12**) showed a similar potency to reduce fatty acid uptake as the positive control nonivamide, reaching 108% inhibition of nonivamide (Figure 2). In addition, a previous study reported the total inhibitory potential of 100 µM capsaicin with −51% in a similar range as well [12]. Next, it was investigated whether positioning of the branching has an impact on the bioactivity of the test compounds. However, fatty acid uptake in Caco-2 cells was not influenced by the position of the methyl-branching at C1 (−38%) (**11**) or C2 (−32%) (**14**) with a total length of the alkyl chain of C3 (*p* < 0.05). Moreover, with a total length of C4, fatty acid uptake was not different when incubated with compound (−17%) (**15**), which has a methyl-branching at the end of the carbon chain at C3, in comparison to compound (**16**), with a methyl-group at C2 (−21%), (*p* < 0.05, Figure 2). 

Since the methyl-branching seemed to be a decisive factor for reducing fatty acid uptake, impact of ethyl-branching at position C1 or C2 at a C4 alkyl chain length was evaluated. Ethyl-branching is leading to a larger molecular volume and shape than methyl-branching. In comparison to the methyl-branched compound (**16**), the ethyl‑branched compound (**18**), increased fatty acid uptake inhibition by 17%. The total inhibitory potential for both tested acetyl-branched compounds in comparison to the control (**17**–**18**) was −38% (Figure 2). These results demonstrate that a higher molecular volume is advantageous for inhibiting fatty acid uptake. A possible explanation for the increased potency of branched-chain molecules could be a facilitated interference with receptors [20]. Candidate receptors in the case of intestinal fatty acid uptake include fatty acid transport proteins, fatty acid binding proteins or CD36. 

Next, the impact of unsaturation of the alkyl chain was investigated. In general, double bonds are associated with a decrease in flexibility and an increase in rigidity [20]. The impact of double-bonds in the fatty acid chain of the ester compounds was tested with three example compounds. Comparison of a hexyl-residue (−8.6%) in ester (**6**) with a 2-*E*-hexenyl-residue (−3.5%) in derivative (**19**) or a 3-*Z*-hexenyl-residue (−9.7%) in substance (**20**) showed no difference in the fatty acid uptake by Caco-2 cells, all three compounds did not significantly decrease fatty acid uptake in comparison to the control (*p* > 0.5). In addition, a 3‑methylbut-2-enyl-residue in compound (**21**) was compared to an isopentyl-residue in ester (**15**), but revealed no difference in fatty acid uptake inhibition as well. Incubation with compound (**21**) decreased fatty acid uptake by 19%, and compound (**15**) by 17% compared to non-treated control cells. 

Removal of the methoxy-group (R2, Figure 3) led to the *p*-hydroxy-derivative (**22**), which, in contrast to replacing it with a second hydroxyl-group like in example (**23**)**,** increased the activity to −21% in comparison to −9% by compound (**4**) or −15% by compound (**23**). In general, these modifications lead to a difference in the lipophilicity of the molecules. However, no correlation of a molecule’s activity and SlogP as a marker for the lipophilicity was detected using Pearson product moment correlation (R = 0.083, *p* > 0.5). Thus, steric reasons rather than a difference in SlogP are assumed to be the cause for the increase in activity by removal of the methoxy-group, which needs to be validated in future studies. 

Finally, also capsiate (**24**) was analyzed, however, in contrast to our initial hypothesis, no activity on fatty acid uptake was detected. It has to be noticed, that capsiate is the direct *O*-analog of capsaicin, at which, in contrast to the homovanillic acid esters tested here, the acid-part is coupled to the alkyl chain, corresponding to a nonclassical isostere of the ester group. Since the alkyl chain length of capsiate with nine carbon atoms should be in the optimal range, and capsiate also bears a methyl branching at the end of the carbon chain, a decisive structural role for the bioisosterism of the ester bond is assumed. 

Moreover, in addition to the mentioned SlogP, also other physiochemical and molecular descriptors were calculated using the RD Kit node on KNIME analytics platform 3.7. The descriptors that showed a considerable difference between the tested compounds, namely SLogP, molecular refractivity (SMR), Labute’s approximate surface area (ASA), molecular weight, number of double bonds, heavy atoms, atoms, and rotable bonds were used for a Pearson product moment correlation analysis. However, none of the mentioned descriptors significantly correlated with the potential to reduce fatty acid uptake (*p* > 0.05), with correlation coefficients of R = 0.083 for SLogP, R = 0.084 for molecular refractivity (SMR), R = 0.080 for Labute’s approximate surface area (ASA), R = 0.079 molecular weight, R = 0.078 for the number of double bonds, R = 0.087 for the number of heavy atoms, R = 0.046 for the number of atoms, and R = 0.2 for the number of rotable bonds.

To summarize the structural analysis of the synthesized homovanillic acid esters, branching of the alkyl chain increased the activity of the test compounds, and, independently from the length, 1-methylated compounds seem more active. In addition, a lack of the methoxy-group at the vanillyl-residue led to an increase of the inhibitory potential for compounds with C4 side chain. In contrast, introduction of double bonds did not have an impact on the inhibitory potential of the test compounds. The present study showed that homovanillic acid esters with a medium chained, branched side chain can reach similar activity as the *N*-alkamide nonivamide, which was used as a positive control. Regarding the stability of homovanillic acid esters when orally ingested, a similar stability as for capsiate, the *O*-analog of capsaicin, is assumed. Although digestion-vulnerable, capsiate possesses similar anti-obesity potential as the amide capsaicin and nonivamide [21,22]. Pharmacokinetic studies failed to detect capsiate in the portal circulation [23], which has been assigned to its instability. The main primary action site for capsiate is thought to be the gastric and the intestinal mucosa [22]. Thus, we hypothesize that homovanillic acid esters could target intestinal fatty acid uptake in the mucosa, directly modulating the function of proteins regulating fatty acid uptake when orally ingested. 

## 3. Experimental Procedures

### 3.1. Chemistry Section

General Information. All chemicals were purchased from commercial suppliers or produced by Symrise AG (Holzminden, Germany) and used without further purification or drying. Capsiate was synthesized according to Scheme S1 (Supplementary Materials). 

Analytical thin-layer chromatography (TLC) was performed on silica-gel GF254 plates with either UV detection at 254 nm or by use of a staining reagent consisting of ammonium molybdate, cerium sulfate, and sulfuric acid. 

Silica gel 60 (0.04–0.063 mm) was used for column chromatography.

^1^H and ^13^C nuclear magnetic resonance spectra were recorded using 5 mm Probes on a Varian Mercury plus Spectrometer (B0 = 9.4 T, Varian, Inc., Palo Alto, CA, USA), a Bruker Ascend spectrometer (B0 = 9.4 T, Bruker Biospin GmbH, Rheinstetten, Germany) or a Bruker Ascend spectrometer (B0 = 14.1 T, Bruker Biospin GmbH, Rheinstetten, Germany), respectively. CDCl3 was used as solvent and tetramethylsilane (TMS) was added to each sample as a chemical shift reference. Chemical shifts (δ) are given in parts per million (ppm). Splitting patterns have been described as follows: s = singlet; d = doublet; t = triplet; q = quartet; dd = double doublet; sext = sextet; hept = heptet; m = multiplet.

GC/MS-Analysis was conducted using a GC-2010 (Shimadzu, Kyoto, Präfektur Kyoto, Japan) fitted with a ZB-1ms capillary column (Phenomenex, Torrance, CA, USA) (20 m × 0.18 mm i.d., df 0.18 µm), coupled with an QP2010 (Shimadzu, Kyoto, Präfektur Kyoto, Japan). The GC conditions for the GC/MS-analysis were: Split injection (split ratio 60:1), injector temperature 60 °C; initial oven temperature at 60 °C for 1 min, ramp at 9 °C/min to 300 °C for 12 min. Helium was used as the carrier gas and the flow rate was 1.0 mL/min.

LC/MS-Analysis and high-resolution masses (for new compounds according to SciFinder) were recorded using a system of LC Waters Acquity UPLC combined with a MS Bruker micrOTOF Q-II. As column a Kinetex RP-C18, 1.7 μm (100 mm × 2.1 mm) was installed (mobile phase: A. H_2_O + 0.1% formic acid; B. acetonitrile + 0.09% Formic acid; flow rate 0.55 mL/min; modus: gradient; temperature: 50 °C; injection volumen: 2 μL; UV range 200–500 nm; LC/MS source ESI+; scan range 50–1600 Dalton).

### 3.2. Synthesis

#### 3.2.1. Method A

Dean-Stark apparatus. Homovanillic acid (1.5–3 g) and the respective alcohol (equimolar) were dissolved in toluene (100 mL), a catalytic amount (1–2 drops) of conc. sulfuric acid was added and the reaction mixture was boiled for 5 h. After washing with sat. aq. NaHCO_3_ solution and water (2×) or optionally sat. aq. NaCl solution, the solvent was removed in vacuo. The product was obtained after purification by column chromatography on silica in about 70% yield.

#### 3.2.2. Method B

Excess of the corresponding alcohol. Homovanillic acid (1.5–3 g) was stirred in the respective alcohol (100 mL) with 0.2–0.5 equivalents of sulfuric acid for 7 h at 90 °C (heating block temperature). Most of the alcohol was removed in vacuo, then sat. aq. NaHCO_3_ solution and EtOAc were added. The organic phase was separated and the aqueous phase extracted with EtOAc. The combined organic layer was washed with sat. aq. NaHCO_3_ solution and water or alternatively with sat. aq. NaCl solution, dried over NaSO_4_ and the solvent removed in vacuo. The product was obtained after purification by column chromatography on silica in 90% to quantitative yield.

#### 3.2.3. Method C

Transesterification of methyl or ethyl homovanillate. Ethyl or methyl homovanillate (5 g) was heated with 2.3 equivalents of respective alcohol and 0.1 equivalents of 25% sodium methylate solution in MeOH to 150–170 °C. At a temperature of 130 °C the atmosphere was lowered subsequently (500 to 50 mbar) and MeOH/EtOH distilled from the reaction mixture over a 1–3 h time period. After dilution with MTBE, the mixture was washed with sat. aq. NH_4_Cl solution and water and the solvent evaporated in vacuum. Excess of respective alcohol was separated by fractional distillation to obtain the crude product (0.1 mbar, subsequent heating to 250 °C). The pure product was obtained after purification by column chromatography on silica in up to 40% yield.

*Methyl-2-(4-hydroxy-3-methoxy-phenyl)acetate* (**1**, method B). ^1^H-NMR (400 MHz, CDCl_3_): *δ* = 6.86 (d, *J* = 8.0 Hz, 1H), 6.80 (d, *J* = 1.9 Hz, 1H), 6.76 (ddq, *J* = 8.0, 2.0, 0.5 Hz, 1H), 5.63–5.59 (m, 1H), 3.877 (s, 3H), 3.69 (s, 3H), 3.547 (t, *J* = 0.6 Hz, 2H). ^13^C-NMR (100 MHz, CDCl_3_): *δ* = 172.4, 146.5, 144.8, 125.7, 122.1, 114.4, 111.7, 55.9, 52.0, 40.8. GCMS: *m*/*z* (%) = 196 [M^+^] (31), 137 (100), 122 (12), 107 (3), 94 (9), 77 (2), 65 (3), 51 (2), 39 (3).

*Ethyl-2-(4-hydroxy-3-methoxy-phenyl)acetate* (**2**, method B). ^1^H-NMR (400 MHz, CDCl_3_): *δ* = 6.85 (d, *J* = 8.05 Hz, 1H), 6.81 (d, *J* = 1.94 Hz, 1H), 6.76 (ddd, *J* = 8.01, 1.99, 0.50 Hz, 1H), 5.61 (d, *J* = 0.36 Hz, 1H), 4.15 (q, *J* = 7.13 Hz, 2H), 3.88 (s, 3H), 3.53 (d, *J* = 0.57 Hz, 2H), 1.25 (t, *J* = 7.13 Hz, 1H). ^13^C-NMR (100 MHz, CDCl_3_): *δ* = 172.0, 146.5, 144.7, 125.9, 122.1, 114.4, 111.7, 60.8, 55.9, 41.0, 14.2. GCMS: *m*/*z* (%) = 210 [M^+^] (30), 137 (100), 122 (11), 107 (2), 94 (8), 77 (2), 66 (3), 51 (3), 39 (3), 29 (8).

*Propyl-2-(4-hydroxy-3-methoxy-phenyl)acetat* (**3**, method B). ^1^H-NMR (400 MHz, CDCl_3_): *δ* = 6.85 (d, *J* = 8.0 Hz, 1H), 6.81 (d, *J* = 2.0 Hz, 1H), 6.76 (dd, *J* = 8.1, 2.0 Hz, 1H), 5.61–5.58 (m, 1H), 4.05 (t, *J* = 6.7 Hz, 2H), 3.88 (s, 3H), 3.54 (s, 2H), 1.71–1.52 (m, 2H), 0.91 (t, *J* = 7.4 Hz, 3H). ^13^C-NMR (100 MHz, CDCl_3_): *δ* = 172.0, 146.5, 144.7, 126.0, 122.2, 144.3, 111.7, 66.4, 55.9, 41.1, 22.0, 10.4. GCMS: *m*/*z* (%) = 224 [M^+^] (30), 137 (100), 122 (10), 107 (2), 94 (8), 77 (2), 66 (3), 51 (2) 43 (8).

*Butyl-2-(4-hydroxy-3-methoxy-phenyl)acetate* (**4**, method B). ^1^H-NMR (400 MHz, CDCl_3_): *δ* = 6.85 (d, *J* = 8.1 Hz, 1H), 6.81 (dd, *J* = 2.0, 0.5 Hz, 1H), 6.76 (ddt, *J* = 8.1, 1.9, 0.6 Hz, 1H), 5.60 (s, 1H), 4.09 (t, *J* = 6.7 Hz, 2H), 3.87 (d, *J* = 0.3 Hz, 3H), 3.53 (t, *J* = 0.5 Hz, 2H), 1.67–1.55 (m, 2H), 1.42–1.29 (m, 2H), 0.91 (t, *J* = 7.4 Hz, 3H). ^13^C-NMR (100 MHz, CDCl_3_): *δ* = 172.0, 146.5, 144.7, 126.0, 122.1, 114.3, 111.7, 64.7, 55.9, 41.1, 30.6, 19.1, 13.7. GCMS: *m*/*z* (%) = 238 [M^+^] (27), 182 (2), 137 (100), 122 (9), 107 (2), 94 (5), 77 (2), 66 (2), 57 (4), 41 (5), 29 (8).

*Pentyl-2-(4-hydroxy-3-methoxy-phenyl)acetat* (**5**, method A). ^1^H-NMR (400 MHz, CDCl_3_): *δ* = 6.85 (d, *J* = 8.0 Hz, 1H), 6.81 (d, *J* = 2.0 Hz, 1H), 6.76 (dd, *J* = 8.1, 2.0 Hz, 1H), 5.58 (s, 1H), 4.08 (t, *J* = 6.7 Hz, 2H), 3.88 (s, 3H), 3.53 (s, 2H), 1.61 (q, *J* = 6.7 Hz, 2H), 1.37–1.24 (m, 4H), 0.94–0.83 (m, 3H). ^13^C-NMR (100 MHz, CDCl_3_): *δ* = 172.0, 146.5, 144.7, 126.0, 122.1, 114.3, 111.7, 65.0, 55.9, 41.1, 28.3, 28.0, 22.3, 14.0. GCMS: *m*/*z* (%) = 252 [M^+^] (28), 182 (5), 137 (100), 122 (10), 94 (5), 66 (3), 43 (13), 29 (4). HRMS (ESI+): calcd. for C_14_H_21_O_4_ [M + H] 253.1434; found 253.1431.

*Hexyl-2-(4-hydroxy-3-methoxy-phenyl)acetate* (**6**, method A). ^1^H-NMR (400 MHz, CDCl_3_): *δ* = 6.85 (dd, *J* = 8.1, 0.3 Hz, 1H), 6.81 (d, *J* = 2.0 Hz, 1H), 6.76 (ddq, *J* = 8.1, 2.0, 0.5 Hz, 1H), 5.58 (s, 1H), 4.08 (t, *J* = 6.7 Hz, 2H), 3.88 (s, 3H), 3.53 (s, 2H), 1.65–1.56 (m, 2H), 1.36–1.20 (m, 6H), 0.87 (t, *J* = 7.0 Hz, 3H). ^13^C-NMR (100 MHz, CDCl_3_): *δ* = 172.0, 146.5, 144.7, 126.0, 122.1, 114.3, 111.7, 65.0, 55.9, 41.1, 31.4, 28.6, 25.5, 22.5, 14.0. GCMS: *m*/*z* (%) = 266 [M^+^] (30), 182 (8), 137 (100), 122 (8), 107 (2), 94 (4), 77 (2), 66 (2), 55 (3), 43 (13).

*Heptyl-2-(4-hydroxy-3-methoxy-phenyl)acetate* (**7**, method A). ^1^H-NMR (400 MHz, CDCl_3_): *δ* = 6.85 (dd, *J* = 8.1, 1.1 Hz, 1H), 6.81 (t, *J* = 1.4 Hz, 1H), 6.76 (dt, *J* = 8.2, 1.4 Hz, 1H), 5.59 (d, *J* = 1.1 Hz, 1H), 4.08 (td, *J* = 6.8, 1.1 Hz, 2H), 3.88 (d, *J* = 1.2 Hz, 3H), 3.53 (s, 2H), 1.68–1.55 (m, 2H), 1.28 (td, *J* = 9.8, 9.3, 4.0 Hz, 8H), 0.94–0.82 (m, 3H). ^13^C-NMR (100 MHz, CDCl_3_): *δ* = 172.0, 146.5, 144.7, 126.0, 122.1, 114.3, 111.7, 65.0, 55.9, 41.1, 31.7, 28.9, 28.6, 25.8, 22.6, 14.1. GCMS: *m*/*z* (%) = 280 [M^+^] (34), 182 (12), 137 (100), 122 (8), 94 (5), 57 (11), 41 (8).

*Octyl-2-(4-hydroxy-3-methoxy-phenyl)acetate* (**8**, method A). ^1^H-NMR (400 MHz, CDCl_3_): *δ* = 6.85 (d, *J* = 8.0 Hz, 1H), 6.81 (dt, *J* = 2.0, 0.4 Hz, 1H), 6.76 (ddq, *J* = 8.5, 2.0, 0.5 Hz, 1H), 5.60 (s, 1H), 4.08 (t, *J* = 6.7 Hz, 2H), 3.87 (s, 3H), 3.53 (s, 2H), 1.65–1.54 (m, 2H), 1.36–1.21 (m, 10H), 0.88 (t, *J* = 7.0 Hz, 3H). ^13^C-NMR (100 MHz, CDCl_3_): *δ* = 172.0, 146.5, 144.7, 125.9, 122.1, 114.4, 111.7, 65.0, 55.9, 41.1, 31.8, 29.2 (2C), 28.6, 25.9, 22.6, 14.1. GCMS: *m*/*z* (%) = 294 [M^+^] (40), 182 (12), 137 (100), 122 (8), 94 (3), 71 (2), 57 (8), 43 (9).

*Decyl-2-(4-hydroxy-3-methoxy-phenyl)acetate* (**9**, method A). ^1^H-NMR (400 MHz, CDCl_3_): *δ* = 6.85 (d, *J* = 8.1 Hz, 1H), 6.81 (d, *J* = 2.0 Hz, 1H), 6.76 (ddq, *J* = 8.1, 1.9, 0.4 Hz, 1H), 5.59 (s, 1H), 4.07 (t, *J* = 6.7 Hz, 2H), 3.88 (s, 3H), 3.53 (s, 2H), 1.65–1.56 (m, 2H), 1.36–1.20 (m, 14H), 0.88 (t, *J* = 7.0 Hz, 3H). ^13^C-NMR (100 MHz, CDCl_3_): *δ* = 172.0, 146.5, 144.7, 125.9, 122.1, 114.3, 111.7, 65.0, 55.9, 41.1, 31.9, 29.5, 29.3 (2C), 29.2, 28.6, 25.9, 22.7, 14.1. GCMS: *m*/*z* (%) = 322 [M^+^] (38), 182 (12), 137 (100), 122 (7), 94 (3), 69 (2), 57 (8), 43 (11).

*Isopropyl-2-(4-hydroxy-3-methoxy-phenyl)acetate* (**10**, method B). ^1^H-NMR (400 MHz, CDCl_3_): *δ* = 6.85 (d, *J* = 8.1 Hz, 1H), 6.81 (dd, *J* = 2.0, 0.5 Hz, 1H), 6.76 (ddt, *J* = 8.0, 2.0, 0.6 Hz,1H), 5.60 (s, 1H), 5.01 (hept, *J* = 6.3 Hz, 1H), 3.88 (s, 3H), 3.496 (t, *J* = 0.5 Hz, 2H), 1.23 (d, *J* = 6.3 Hz, 6H). ^13^C-NMR (100 MHz, CDCl_3_): *δ* = 171.5, 146.4, 144.7, 126.1, 122.1, 114.3, 111.7, 68.1, 55.9, 41.3, 21.8 (2C). GCMS: *m*/*z* (%) = 224 [M^+^] (30), 137 (100), 122 (10), 107 (2), 94 (6), 77 (3), 66 (5), 51 (3), 43 (15).

*sec-Butyl-2-(4-hydroxy-3-methoxy-phenyl)acetate* (**11**, method B). ^1^H-NMR (400 MHz, CDCl_3_): *δ* = 6.85 (d, *J* = 8.1 Hz, 1H), 6.82 (d, *J* = 1.9 Hz, 1H), 6.79–6.74 (m, 1H), 5.57 (s, 1H), 4.92–4.78 (m, 1H), 3.88 (s, 3H), 3.51 (t, *J* = 0.5 Hz, 2H), 1.62–1.46 (m, 2H), 1.19 (d, *J* = 6.3 Hz, 3H), 0.85 (t, *J* = 7.5 Hz, 3H). ^13^C-NMR (100 MHz, CDCl_3_): *δ* = 171.6, 146.4, 144.7, 126.2, 122.1, 114.3, 111.7, 72.7, 55.9, 41.4, 28.8, 19.4, 9.6. GCMS: *m*/*z* (%) = 238 [M^+^] (30), 137 (100), 122 (8), 107 (2), 94 (5), 77 (2), 66 (3), 57 (20), 51 (2), 41 (8), 29 (8).

*1-Methylpentyl-2-(4-hydroxy-3-methoxy-phenyl)acetate* (**12**, method C). ^1^H-NMR (400 MHz, CDCl_3_): *δ* = 6.85 (d, *J* = 8.1 Hz, 1H), 6.81 (d, *J* = 2.0 Hz, 1H), 6.76 (dd, *J* = 8.1, 2.0 Hz, 1H), 5.55 (s, 1H), 4.90 (h, *J* = 6.8, 6.2 Hz, 1H), 3.88 (s, 3H), 3.53–3.48 (m, 2H), 1.63–1.52 (m, 1H), 1.52–1.42 (m, 1H), 1.32–1.21 (m, 4H), 1.19 (d, *J* = 6.3 Hz, 3H), 0.86 (t, *J* = 7.0 Hz, 3H). ^13^C-NMR (100 MHz, CDCl_3_): *δ* = 171.6, 146.4, 144.7, 126.2, 122.1, 114.3, 111.7, 71.5, 55.9, 41.4, 35.6, 27.5, 22.5, 19.9, 13.9. GCMS: *m*/*z* (%) = 266 [M^+^] (30), 182 (7), 137 (100), 122 (9), 94 (8), 85(9), 77 (2), 66 (3), 57 (8), 43 (35). HRMS (ESI+): calcd. for C_15_H_23_O_4_ [M + H] 267.1591; found 267.1597.

*1-Methylhexyl-2-(4-hydroxy-3-methoxy-phenyl)acetate* (**13**, method C). ^1^H-NMR (400 MHz, CDCl_3_): *δ* = 6.85 (d, *J* = 8.0 Hz, 1H), 6.81 (d, *J* = 1.9 Hz, 1H), 6.76 (dd, *J* = 8.1, 1.9 Hz, 1H), 5.55 (s, 1H), 4.90 (sext, *J* = 6.4 Hz, 1H), 3.88 (s, 3H), 3.50 (s, 2H), 1.62–1.51 (m, 1H), 1.50–1.40 (m, 1H), 1.29–1.21 (m, 6H), 1.19 (d, *J* = 6.3 Hz, 3H), 0.86 (t, *J* = 6.9 Hz, 3H). ^13^C-NMR (100 MHz, CDCl_3_): *δ* = 171.6, 146.4, 144.7, 126.2, 122.1, 114.3, 111.6, 71.5, 55.9, 41.4, 35.9, 31.6, 24.9, 22.5, 19.9, 13.9. GCMS: *m*/*z* (%) = 280 [M^+^] (25), 182 (8), 137 (100), 122 (9), 94 (5), 77 (1), 66 (1), 57 (35), 43 (16). HRMS (ESI+): calcd. for C16H25O4 [M + H] 281.1747; found 281.1762.

*Isobutyl-2-(4-hydroxy-3-methoxy-phenyl)acetate* (**14**, method B). ^1^H-NMR (400 MHz, CDCl_3_): *δ* = 6.85 (dd, *J* = 8.1, 0.3 Hz, 1H), 6.82–6.81 (m, 1H), 6.77 (ddt, *J* = 8.1, 2.0, 0.6 Hz, 1H), 5.60 (s, 1H), 3.87 (d, *J* = 0.3 Hz, 3H), 3.86 (d, *J* = 6.6 Hz, 2H), 3.54 (t, *J* = 0.5 Hz, 2H), 1.91 (dq, *J* = 6.7 Hz, 1H), 0.90 (d, *J* = 6.7 Hz, 6H). ^13^C-NMR (100 MHz, CDCl_3_): *δ* = 172.0, 146.5, 144.7, 126.0, 122.1, 114.3, 111.7, 70.9, 55.9, 41.1, 27.7, 19.0 (2C). GCMS: *m*/*z* (%) = 238 [M^+^] (30), 182 (5), 137 (100), 122 (9), 107 (2), 94 (6), 77 (2), 66 (3), 57 (11), 51 (2), 41 (8), 29 (7). HRMS (ESI+): calcd. for C_13_H_19_O_4_ [M + H] 239.1278; found 239.1275.

*Isopentyl-2-(4-hydroxy-3-methoxy-phenyl)acetate* (**15**, method B). ^1^H-NMR (400 MHz, CDCl_3_): *δ* = 6.85 (d, *J* = 8.0 Hz, 1H), 6.81 (d, *J* = 1.9 Hz, 1H), 6.76 (dd, *J* = 8.1, 1.9 Hz, 1H), 5.57 (s, 1H), 4.11 (t, *J* = 6.8 Hz, 2H), 3.88 (s, 3H), 3.53 (s, 2H), 1.73–1.56 (m, *J* = 6.9 Hz, 1H), 1.51 (q, *J* = 6.9 Hz, 2H), 0.90 (d, *J* = 6.6 Hz, 6H). ^13^C-NMR (100 MHz, CDCl_3_): *δ* = 172.1, 146.6, 144.9, 126.1, 122.3, 114.5, 111.8, 63.7, 56.0, 41.2, 37.4, 25.2, 22.6 (2C). GCMS: *m*/*z* (%) = 252 [M^+^] (26), 182 (11), 137 (100), 122 (9), 94 (8), 77 (2), 66 (3), 55 (5), 43 (15). HRMS (ESI+): calcd. for C_14_H_21_O_4_ [M + H] 253.1434; found 253.1429.

*2-Methylbutyl-2-(4-hydroxy-3-methoxy-phenyl)acetate* (**16**, method B). ^1^H-NMR (400 MHz, CDCl_3_): *δ* = 6.86 (d, *J* = 8.1 Hz, 1H), 6.81 (d, *J* = 2.0 Hz, 1H), 6.77 (dd, *J* = 8.0, 2.0 Hz, 1H), 5.55 (s, 1H), 3.97 (dd, *J* = 10.7, 6.0 Hz, 1H), 3.90 (dd, *J* = 10.8, 6.7 Hz, 1H), 3.88 (s, 3H), 3.54 (s, 2H), 1.69 (dddd, *J* = 12.4, 7.8, 6.8, 5.8 Hz, 1H), 1.38 (dtd, *J* = 13.1, 7.5, 5.6 Hz, 1H), 1.23–1.08 (m, 1H), 0.88 (d, *J* = 6.8 Hz, 3H), 0.88 (t, *J* = 7.5 Hz, 3H). ^13^C-NMR (100 MHz, CDCl_3_): *δ* = 172.0, 146.4, 144.7, 126.0, 122.1, 114.3, 111.7, 69.4, 55.9, 41.1, 34.1, 26.0, 16.3, 11.2. GCMS: *m*/*z* (%) = 252 [M+] (30), 182 (10), 137 (100), 122 (8), 94 (7), 71 (5), 55 (4), 43 (18), 29 (10). HRMS (ESI+): calcd. for C_14_H_21_O_4_ [M + H] 253.1434; found 253.1434.

*1-Ethylbutyl-2-(4-hydroxy-3-methoxy-phenyl)acetate* (**17**, method C). ^1^H-NMR (400 MHz, CDCl_3_): *δ* = 6.85 (d, *J* = 8.0 Hz, 1H), 6.82 (d, *J* = 1.9 Hz, 1H), 6.77 (dd, *J* = 8.1, 2.0 Hz, 1H), 5.56 (s, 1H), 4.83 (tt, *J* = 7.1, 5.3 Hz, 1H), 3.88 (s, 3H), 3.52 (s, 2H), 1.60–1.41 (m, 4H), 1.38–1.17 (m, 2H), 0.87 (t, *J* = 7.4 Hz, 3H), 0.83 (t, *J* = 7.5 Hz, 3H). ^13^C-NMR (100 MHz, CDCl_3_): *δ* = 171.8, 146.4, 144.6, 126.2, 122.1, 114.3, 111.7, 75.7, 55.9, 41.4, 35.8, 26.9, 18.5, 13.9, 9.5. GCMS: *m*/*z* (%) = 266 [M^+^] (25), 182 (5), 137 (100), 85 (8), 57 (5), 43 (25). HRMS (ESI+): calcd. for C_15_H_23_O_4_ [M + H] 267.1591; found 267.1584.

*2-Ethylbutyl-2-(4-hydroxy-3-methoxy-phenyl)acetate* (**18**, method A). ^1^H-NMR (400 MHz, CDCl_3_): *δ* = 6.85 (d, *J* = 8.1 Hz, 1H), 6.81 (d, *J* = 1.9 Hz, 1H), 6.76 (dd, *J* = 8.1, 1.9 Hz, 1H), 5.56 (s, 1H), 4.01 (d, *J* = 5.8 Hz, 2H), 3.88 (s, 3H), 3.53 (s, 2H), 1.49 (h, *J* = 6.2 Hz, 1H), 1.38–1.25 (m, 4H), 0.86 (t, *J* = 7.5 Hz, 6H). ^13^C-NMR (100 MHz, CDCl_3_): *δ* = 10.97 (2C), 23.26 (2C), 40.26, 41.14, 55.86, 66.86, 111.68, 114.29, 122.12, 125.98, 144.67, 146.41, 172.07. GCMS: *m*/*z* (%) = 266 [M^+^] (28), 182 (18), 137 (100), 122 (8), 107 (2), 94 (6), 85 (3), 77 (2), 66 (5), 57 (4), 43 (28). HRMS (ESI+): calcd. for C_15_H_23_O_4_ [M + H] 267.1591; found 267.1599.

*[(E)-Hex-2-enyl]-2-(4-hydroxy-3-methoxy-phenyl)acetate* (**19**, method C). ^1^H-NMR (600 MHz, CDCl_3_): *δ* = 6.86 (d, *J* = 8.0 Hz, 1H), 6.81 (d, *J* = 1.9 Hz, 1H), 6.77 (dd, *J* = 8.1, 1.9 Hz, 1H), 5.74 (dtt, *J* = 14.9, 6.8, 1.3 Hz, 1H), 5.55 (dtt, *J* = 15.3, 6.3, 1.1 Hz, 2H), 4.53 (dd, *J* = 6.5, 1.1 Hz, 2H), 3.88 (s, 3H), 3.55 (s, 2H), 2.02 (q, *J* = 7.3 Hz, 2H), 1.39 (sext, *J* = 7.4 Hz, 2H), 0.89 (t, *J* = 7.4 Hz, 3H). ^13^C-NMR (150 MHz, CDCl_3_): *δ* = 171.7, 146.4, 144.7, 136.5, 125.8, 123.7, 122.1, 114.3, 111.7, 65.7, 55.9, 40.9, 34.3, 22.0, 13.6. LCMS (ESI+): *m*/*z* (%) = 287 (100) [M + Na], 265 (45) [M + H], 195 (67) [C_10_H_11_O_4_], 137 (87) [C_8_H_9_O_2_]. HRMS (ESI+): calcd. for C_15_H_21_O_4_ [M + H] 265.1434; found 265.1429.

*[(Z)-Hex-3-enyl]-2-(4-hydroxy-3-methoxy-phenyl)acetate* (**20**, method A). ^1^H-NMR (400 MHz, CDCl_3_): *δ* = 6.85 (d, *J* = 8.1 Hz, 1H), 6.80 (d, *J* = 2.0 Hz, 1H), 6.76 (dd, *J* = 8.0, 2.0 Hz, 1H), 5.58 (s, 1H), 5.49 (ddt, *J* = 10.9, 7.3, 1.6 Hz, 1H), 5.29 (ddt, J = 10.7, 7.3, 1.5 Hz, 1H), 4.08 (t, *J* = 7.0 Hz, 2H), 3.88 (s, 3H), 3.53 (s, 2H), 2.43–2.32 (m, 2H), 2.03 (pd, *J* = 7.5, 1.6 Hz, 2H), 0.96 (t, *J* = 7.5 Hz, 3H). ^13^C-NMR (100 MHz, CDCl_3_): *δ* = 171.9, 146.5, 144.7, 134.6, 125.8, 123.6, 122.1, 114.3, 111.7, 64.4, 55.9, 41.0, 26.7, 20.6, 14.2. GCMS: *m*/*z* (%) = 264 [M^+^] (30), 182 (55), 137 (100), 122 (15), 94 (10), 82 (8), 67 (15), 55 (20), 41 (15). HRMS (ESI+): calcd. for C_15_H_21_O_4_ [M + H] 265.1434; found 265.1441.

*3-Methylbut-2-enyl-2-(4-hydroxy-3-methoxy-phenyl)acetate* (**21**, method C). ^1^H-NMR (400 MHz, CDCl_3_): *δ* = 6.85 (d, *J* = 8.0 Hz, 1H), 6.81 (d, *J* = 2.0 Hz, 1H), 6.76 (dd, *J* = 8.1, 2.0 Hz, 1H), 5.57 (s, 1H), 5.34 (thept, *J* = 7.3, 1.3 Hz, 1H), 4.59 (d, *J* = 7.2 Hz, 2H), 3.88 (s, 3H), 3.54 (s, 2H), 1.76–1.74 (m, 3H), 1.70–1.68 (m, 3H). ^13^C-NMR (100 MHz, CDCl_3_): *δ* = 171.9, 146.5, 144.7, 139.2, 125.9, 122.1, 118.5, 114.3, 111.7, 61.8, 55.9, 40.9, 25.8, 18.0. GCMS: *m*/*z* (%) = 250 [M^+^] (20), 182 (1), 137 (100), 122 (10), 94 (8), 77 (2), 69 (45), 66 (3), 53 (3), 41 (22). HRMS (ESI+): calcd. for C_14_H_19_O_4_ [M + H] 251.1278; found 251.1288.

*Butyl-2-(4-hydroxyphenyl)acetate* (**22**, method B). ^1^H-NMR (400 MHz, CDCl_3_): *δ* = 7.10 (d, *J* = 8.5 Hz, 2H), 6.72 (d, *J* = 8.5 Hz, 2H), 5.97 (s, 1H, *OH*), 4.10 (t, *J* = 6.7 Hz, 2H), 3.54 (s, 2H), 1.60 (ddt, *J* = 8.8, 7.9, 6.5 Hz, 2H), 1.43–1.26 (m, 2H), 0.91 (t, *J* = 7.4 Hz, 3H). ^13^C-NMR (100 MHz, CDCl_3_): *δ* = 172.8, 154.9, 130.4 (2C), 125.8, 115.5 (2C), 64.9, 40.5, 30.5, 19.0, 13.6. GCMS: *m*/*z* (%) = 208 [M^+^] (15), 152 (5), 107 (100), 77 (10), 57 (9), 41 (8), 29 (10).

*Butyl-2-(3,4-dihydroxyphenyl)acetate* (**23**, method B). ^1^H-NMR (400 MHz, CDCl_3_): *δ* = 6.76–6.73 (m, 1H), 6.72 (s, 1H), 6.64 (dd, *J* = 8.1, 2.1 Hz, 1H), 4.11 (t, *J* = 6.7 Hz, 2H), 3.50 (s, 2H), 1.62 (ddt, *J* = 8.9, 7.9, 6.6 Hz, 2H), 1.42–1.30 (m, 2H), 0.92 (t, *J* = 7.4 Hz, 3H). ^13^C-NMR (100 MHz, CDCl_3_): *δ* = 173.4, 143.8, 143.1, 126.2, 121.7, 116.3, 115.4, 65.2, 40.7, 30.5, 19.0, 13.7. LCMS (ESI+): *m*/*z* (%) = 247 (22) [M + Na], 225 (56) [M + H], 123 (100) [C_7_H_7_O_2_]. HRMS (ESI+): calcd. for C_12_H_17_O_4_ [M + H] 225.1121; found 225.1141.

The corresponding NMR and HRMS spectra of new compounds according to SciFinder are provided in the Supplemental Material (Figures S1–S24).

### 3.3. Biological Evaluation

#### 3.3.1. Cell Culture

Caco-2 cells were obtained from ATCC (Catalog Number: HTB-37, LGC Standards GmbH, Germany), cell culture media (Dulbecco’s modified Eagles medium), PBS, and cell culture-grade supplements from Lonza Cologne GmbH, Cologne, Germany. Caco-2 cells were cultured in DMEM supplemented with 10% FBS, 2% l-glutamine, and 1% Penicillin/Streptomycin at 5% CO_2_ in a humidified incubator and used up to passage 25. Differentiation into an enterocyte-like phenotype was carried out by cultivation for 21 days with normal growth medium in 96-well plates (Corning GmbH, Kaiserlautern, Germany) as described before [12]. Medium was changed every two to three days during differentiation process. Previous results using this method demonstrated transepithelial electrical resistance (TEER) values of >500 Ω/cm^2^ upon differentiation [9,12], demonstrating an intact monolayer and enterocyte-like phenotype [24]. 

#### 3.3.2. In Vitro Fatty Acid Uptake Studies

Uptake of the fatty acid Bodipy-C12 was used to determine fatty acid uptake in differentiated Caco-2 cells using the QBT Fatty Acid Uptake Kit (Molecular Devices, Biberach, Germany) as described before in detail [9,12]. Caco-2 cells were starved for one h in 90 µL serum free DMEM per well at 37 °C before pre-treatment with the test compounds for 30 min at 37 °C. The test compounds were dissolved in ethanol as 1000× stock solutions and further diluted in Hank’s Balanced salt solution (HBSS, Sigma-Aldrich, Taufkirchen, Germany, H6648) supplemented with 20 mM HEPES buffer reagent to reach a 10× concentrated solution, of which 10 µL was added to the respective well. The final concentration of ethanol was 0.1%. As controls, HBSS/HEPES without or with the addition of 0.1% ethanol was tested. In addition, 100 µM of nonivamide was tested as a positive control for each measurement. The fatty acid uptake reaction was started by addition of the loading dye containing Bodipy-C12 coupled to a fluorescence quencher. Uptake of the Bodipy-C12 into the cells removed the quencher and the increasing fluorescence signal was determined using the kinetic mode on a Synergy HT plate reader (Biotek Instruments Inc., Bad Friedrichshall, Germany) for one hour. The area under the curves (AUC) was analyzed from the respective time-intensity-plots and used as the measure for a compounds potential to reduce fatty acid uptake. The lower the AUC, the higher the inhibitory potential of the test compound (Figure 4B). 

#### 3.3.3. Physicochemcial Descriptors

A number of topological descriptors was calculated using the RD Kit (descriptor calculation) node on KNIME analytics platform 3.7, but only those displaying values with considerable difference between the tested molecules were used further correlation analysis. Those descriptors (SlogP, molecular refractivity (SMR), Labute’s approximate surface area, exact molecular weight, number of double bonds, atoms, heavy atoms and rotable bonds) were correlated to the activity levels using Pearson’s product moment correlation with the R Snippet node, which is using R as a back end, on KNIME analytics platform 3.7. 

#### 3.3.4. Statistical Analysis

Data are calculated as the mean AUC ± SD from three independent experiments with two technical replicates per measurement for each compound and concentrations. Data were tested for normal distribution using Shapiro-Wilk Test. A Two-Way ANOVA using Holm-Sidak Post Hoc Test was applied to analyze the data for concentration and compound specific effects, and One-Way ANOVA using Holm-Sidak Post Hoc Test for analyzing differences between the different treatment groups. Statistical analysis was carried out using SigmaPlot 13.0 (Systat Software GmbH, Erkrath, Germany). 

## 4. Conclusions

The present study demonstrated that a medium chained, branched side chain is favorable to reduce intestinal fatty acid uptake by homovanillic acid esters. The presented data serve as an important basis for designing potent dietary measures or to search for natural products with increased potential to reduce intestinal fatty acid uptake. Future studies are needed to evaluate the mechanism of action, dose-dependency of the most potent compound, and whether these results may be transferred to other classes of compounds.

## Supplementary Materials

The supplementary materials are available.
