# Supplementary material for: Biological Evaluation of Natural and Synthesized Homovanillic Acid Esters as Inhibitors of Intestinal Fatty Acid Uptake in Differentiated Caco-2 Cells
Source: Molecules. 2019 Oct 7;24(19):3599. doi: 10.3390/molecules24193599 (PMC6803983; doi:10.3390/molecules24193599)
Supplement: Supplementary file 1 [file molecules-24-03599-s001.pdf]

Biological evaluation of natural and synthesized homovanillic acid esters as inhibitors of intestinal fatty acid uptake in differentiated Caco-2 cells

Supplemental information

Synthesis of capsiate

The known substrate Capsiate (**24**) was synthesized according to literature procedures or using common general synthesis methods (scheme **S1**). The used building blocks **m** and **p** were synthesized starting from vanillin (**n**) and isobutyraldehde (**a**).

Building block **m**: First isobutylaldehyde (**a**) was converted to enol **c** using vinylmagnesium bromide (**b**) in THF. With triethyl orthoacetate **d** in propionic acid the ethyl ester **e** was generated under reflux conditions. This ester **e** was reduced by $LiAlH_4$ in ether to the corresponding alcohol **f** and mesyl protected to compound **g** [*Tetrahedron* **1996**, *52*, 8451–8470].

Condensation of dimethyl malonate **h** with mesylate **g** in methanol followed by NaCl treatment in DMSO afforded methyl ester **k** which in turn was saponified using potassium hydroxide in MeOH/water (1:1). Acid **l** was converted into its acid chloride **m** by oxalyl chloride in dichloromethane.

Building block **p**: Vanillin (**n**) was TBDMS protected using TBDMS chloride in dichloromethane and the resulting compound **o** was afterwards reduced to the alcohol **p** with $LiAlH_4$ in THF.

Finally, esterification of acid chloride **m** with alcohol **p** in pyridine followed by TBDMS (*tert*-butyl dimethylsilyl) deprotection using TBAF (tetra-*n*-butylammonium fluoride) in THF afforded the capsiate (24).

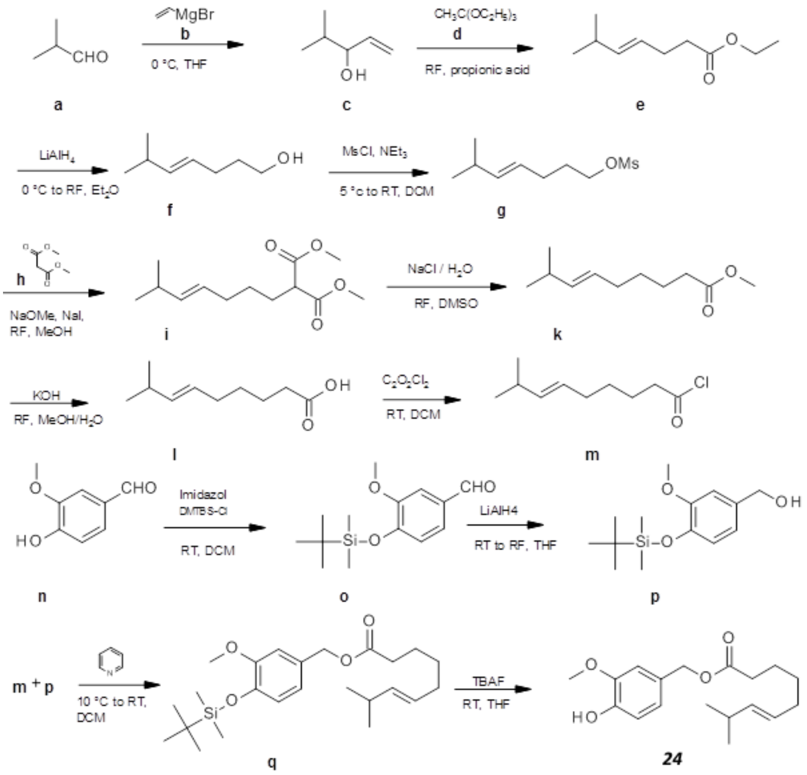

Scheme **S1**.

[1] *Tetrahedron* **1996**, *52*, 8451–8470.

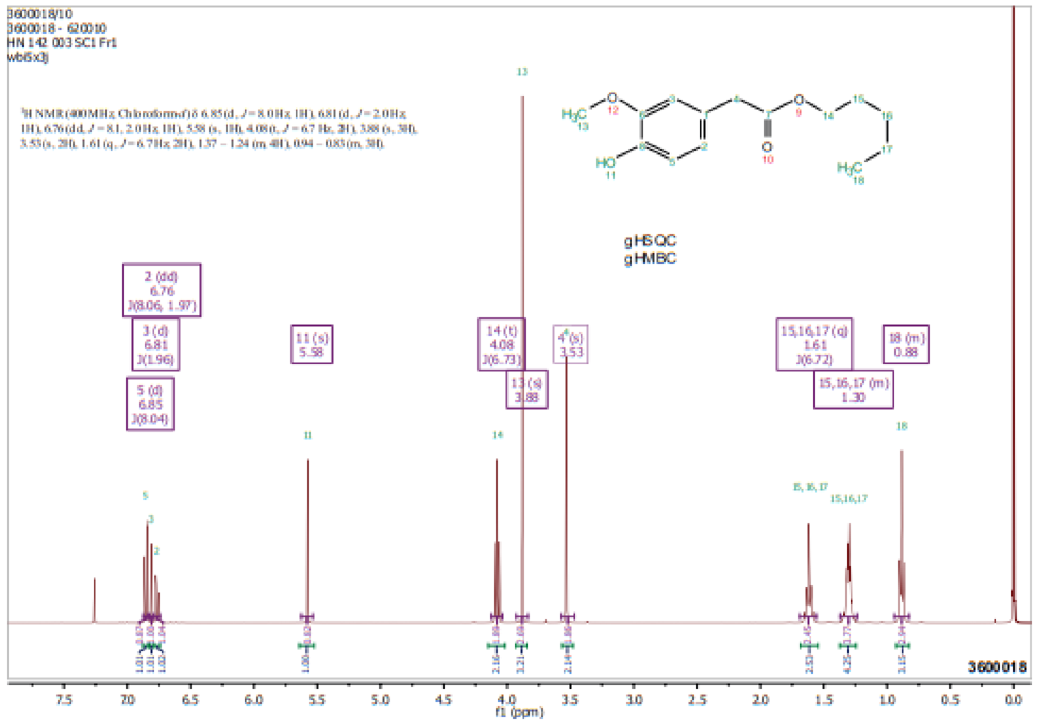

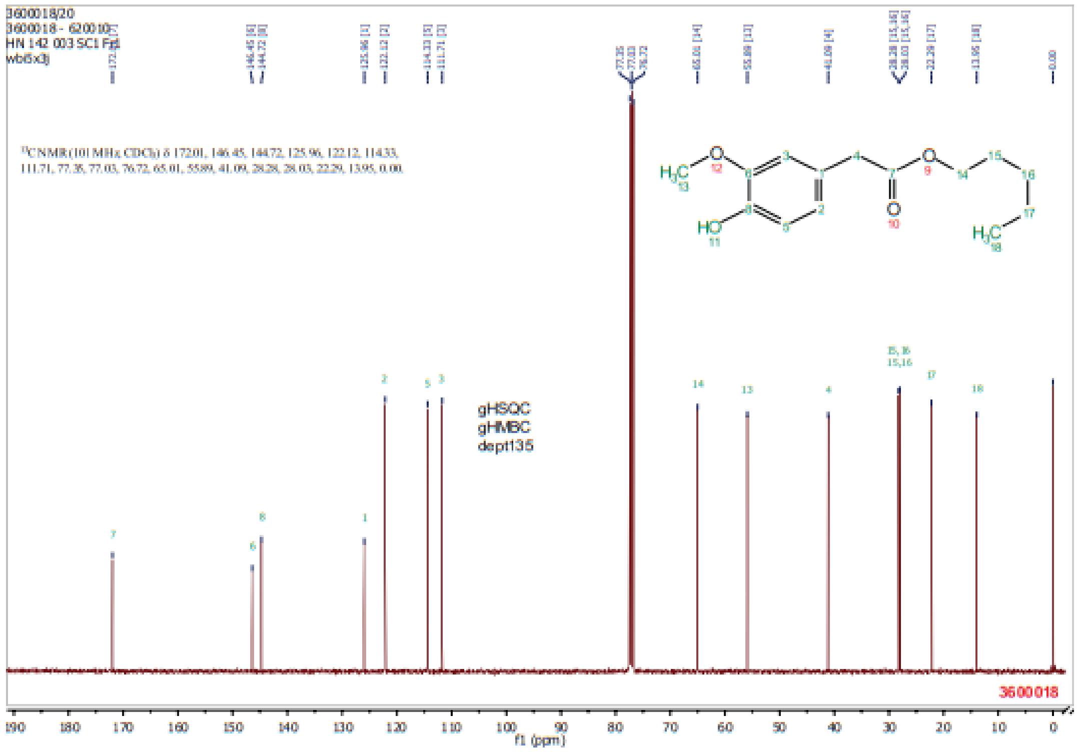

Figure S1: ^{1}H NMR and ^{13}C NMR Compound 5

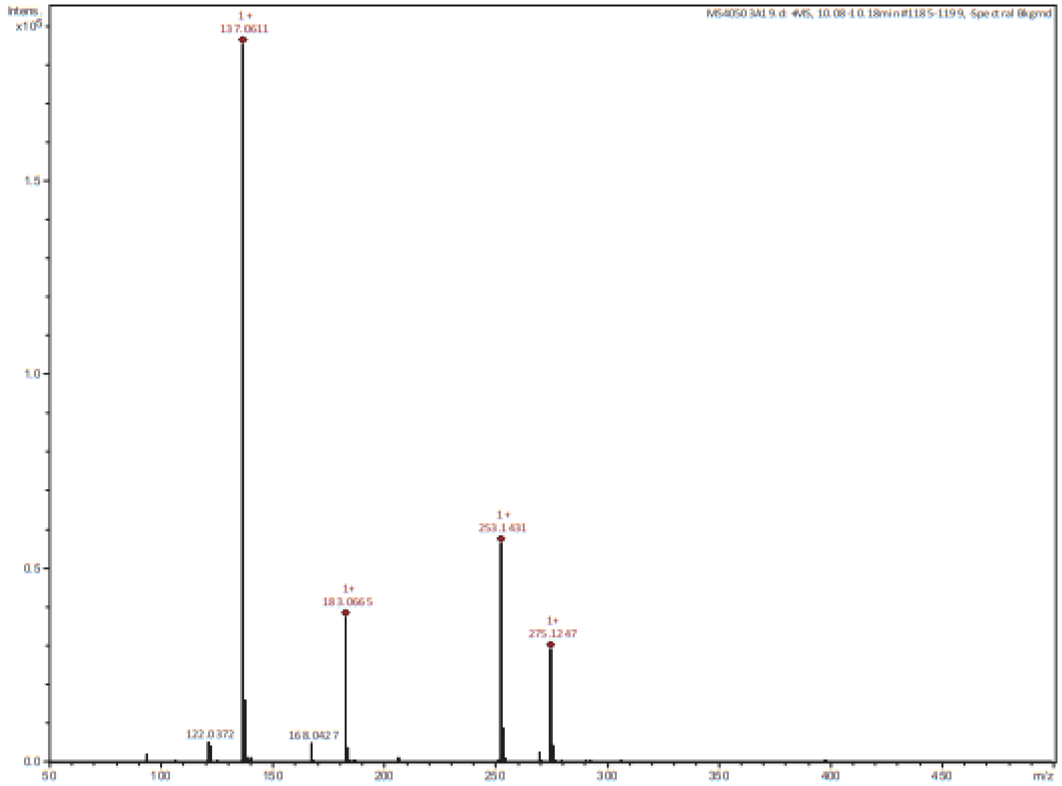

Figure S2: HR-ESIMS Compound 5

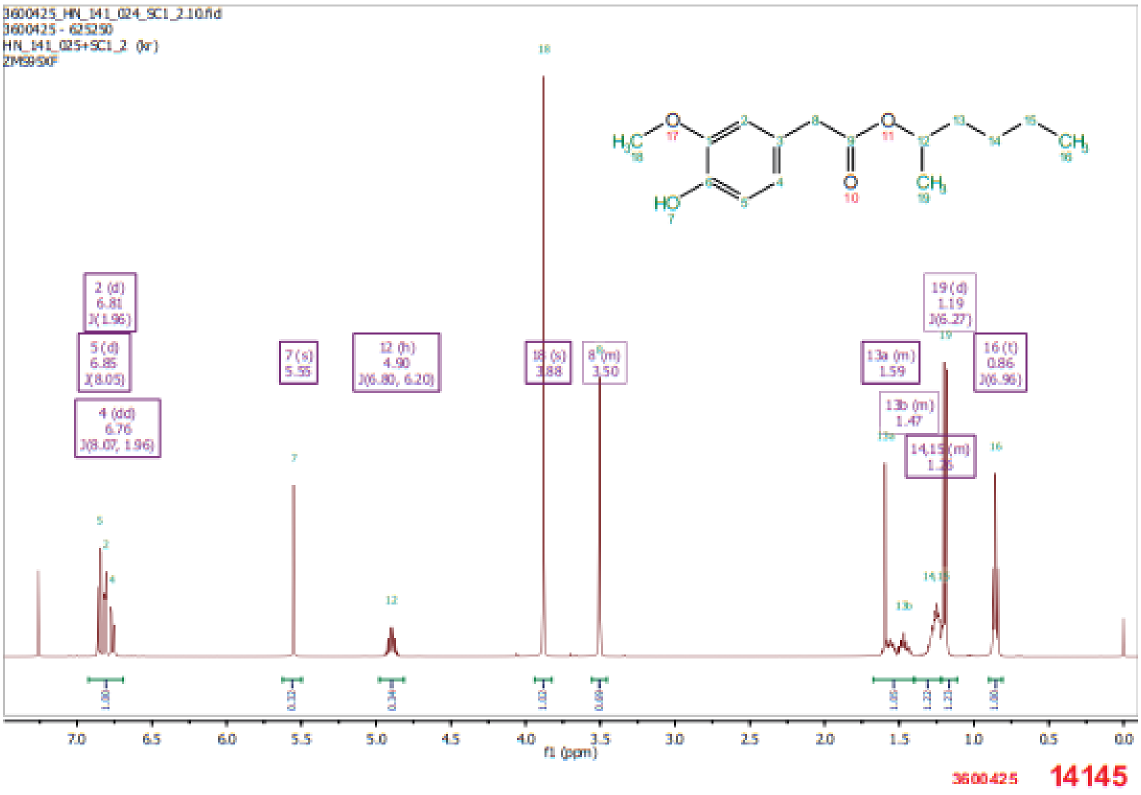

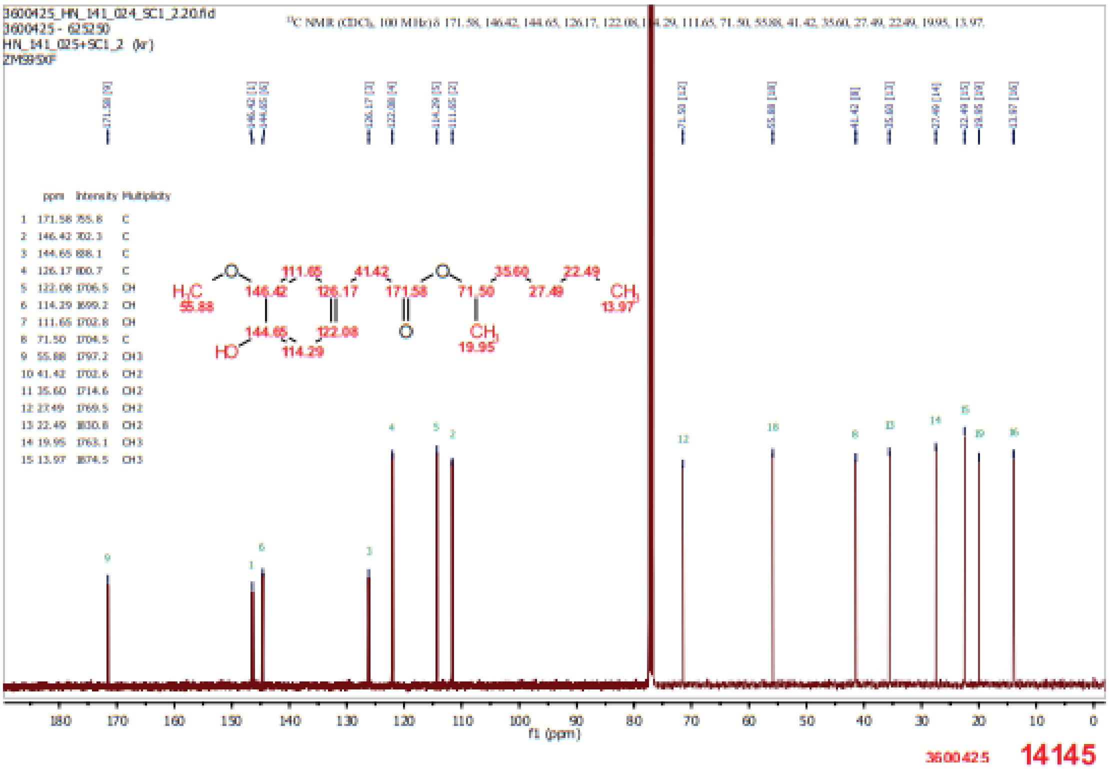

Figure S3: ^{1}H NMR and ^{13}C NMR Compound 12

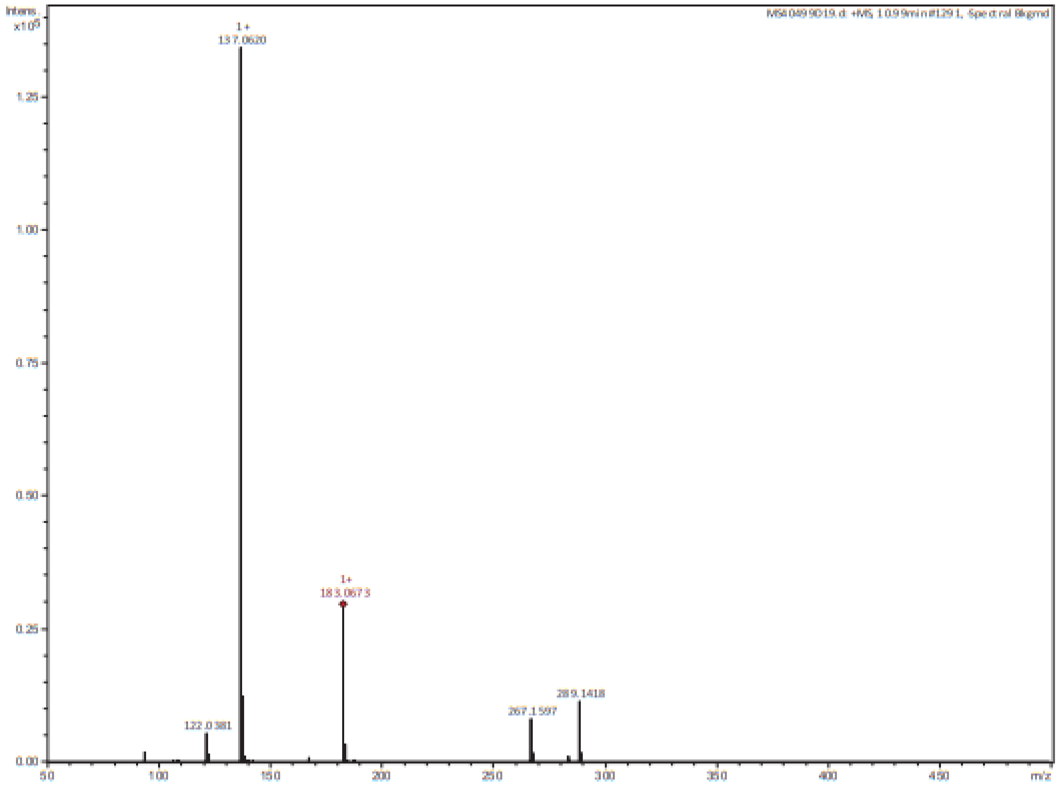

Figure S4: HR-ESIMS Compound 12

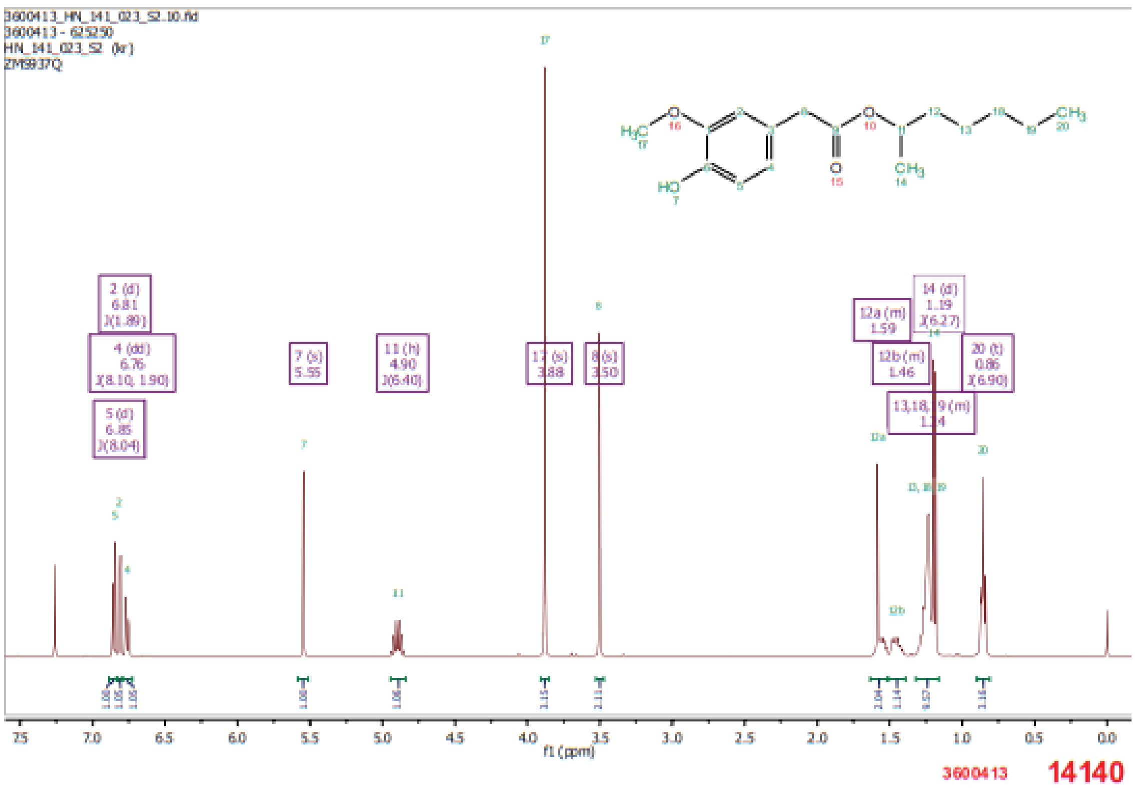

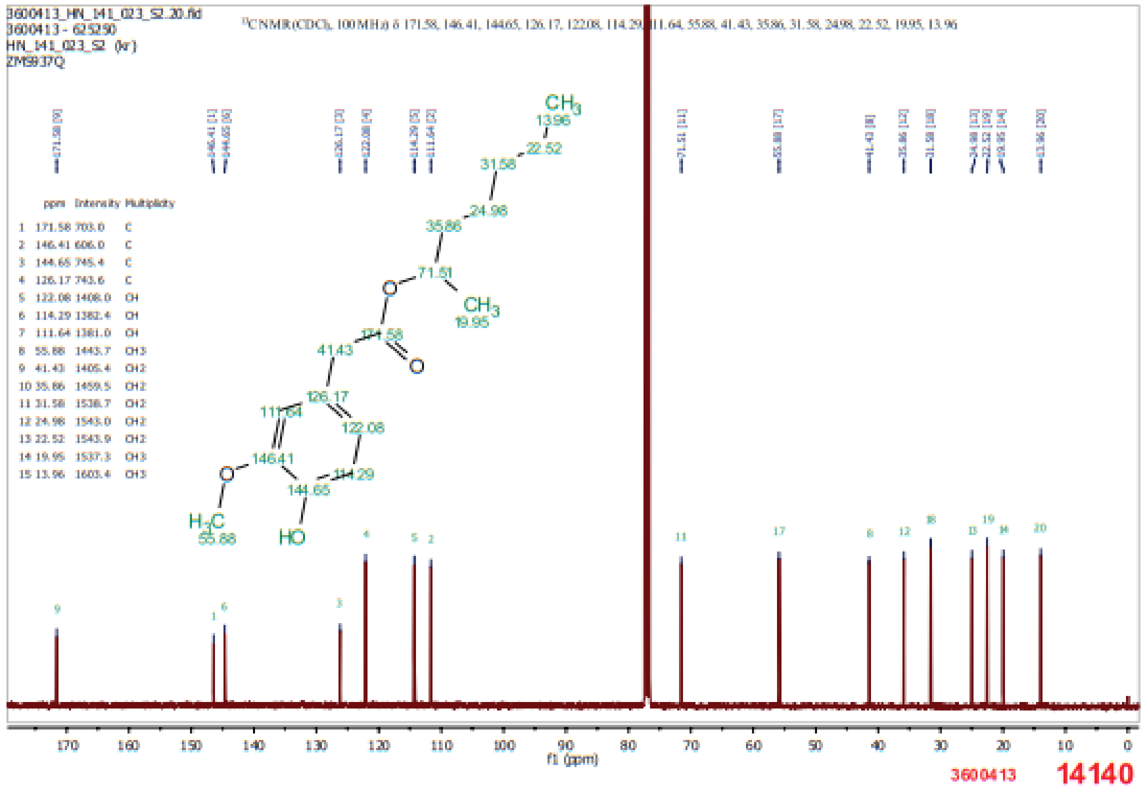

Figure S5: ^{1}H NMR and ^{13}C NMR Compound 13

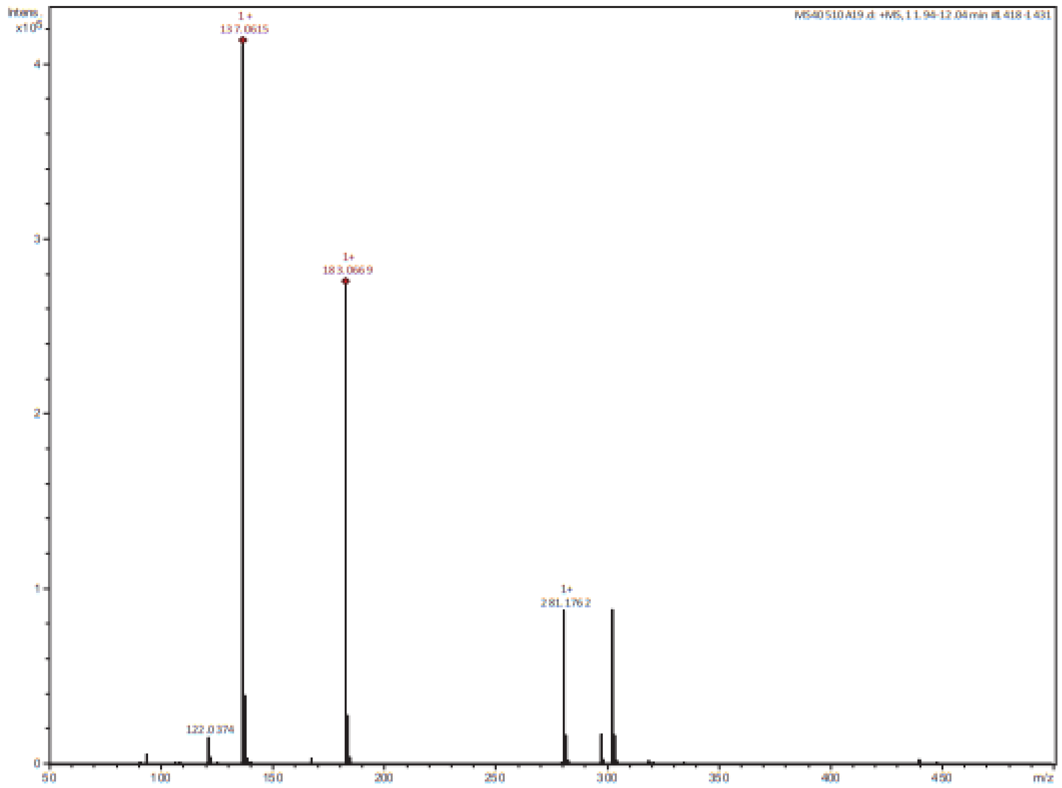

Figure S6: HR-ESIMS Compound 13

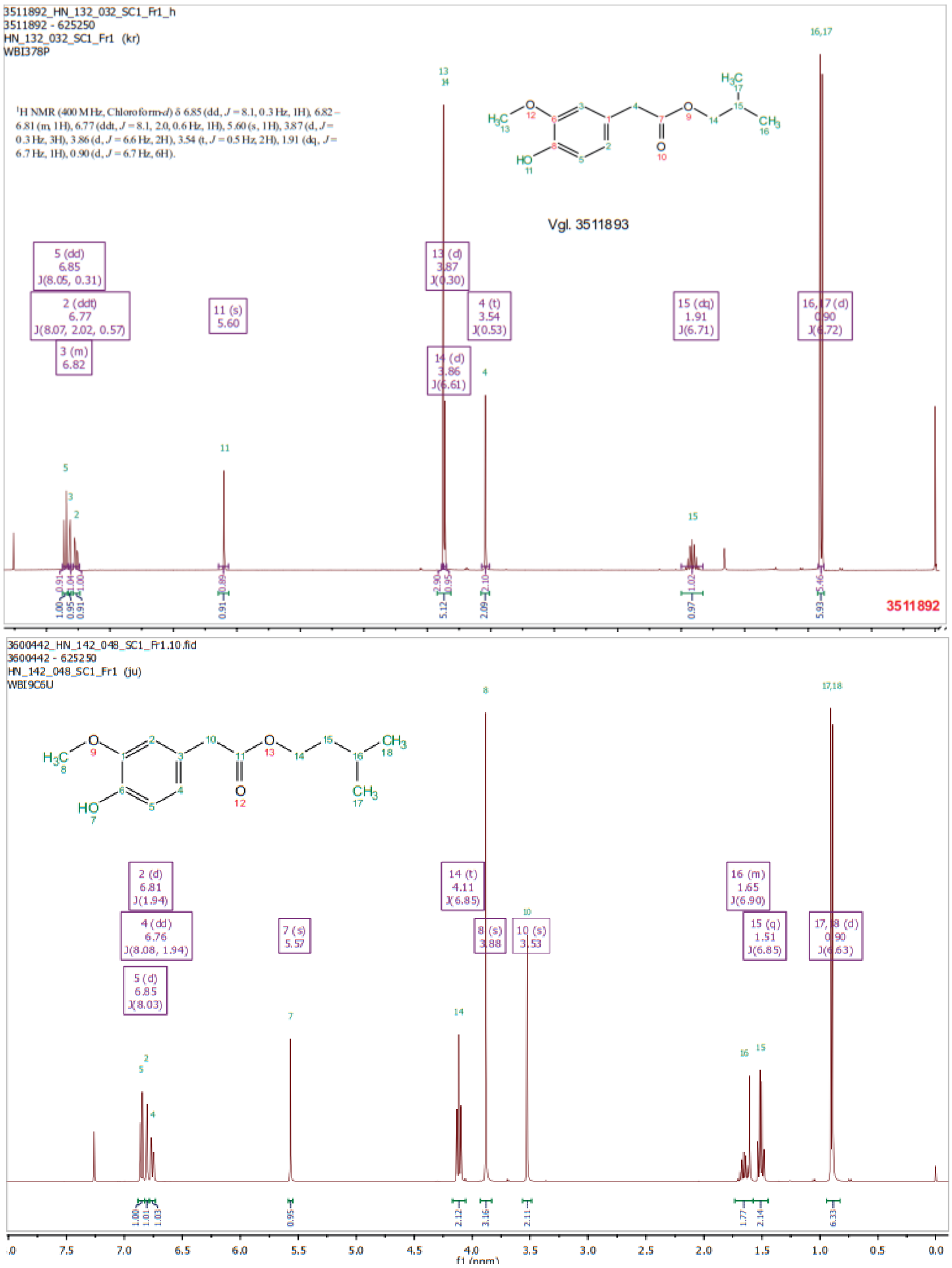

Figure S7: ^{1}H NMR and ^{13}C NMR Compound 14

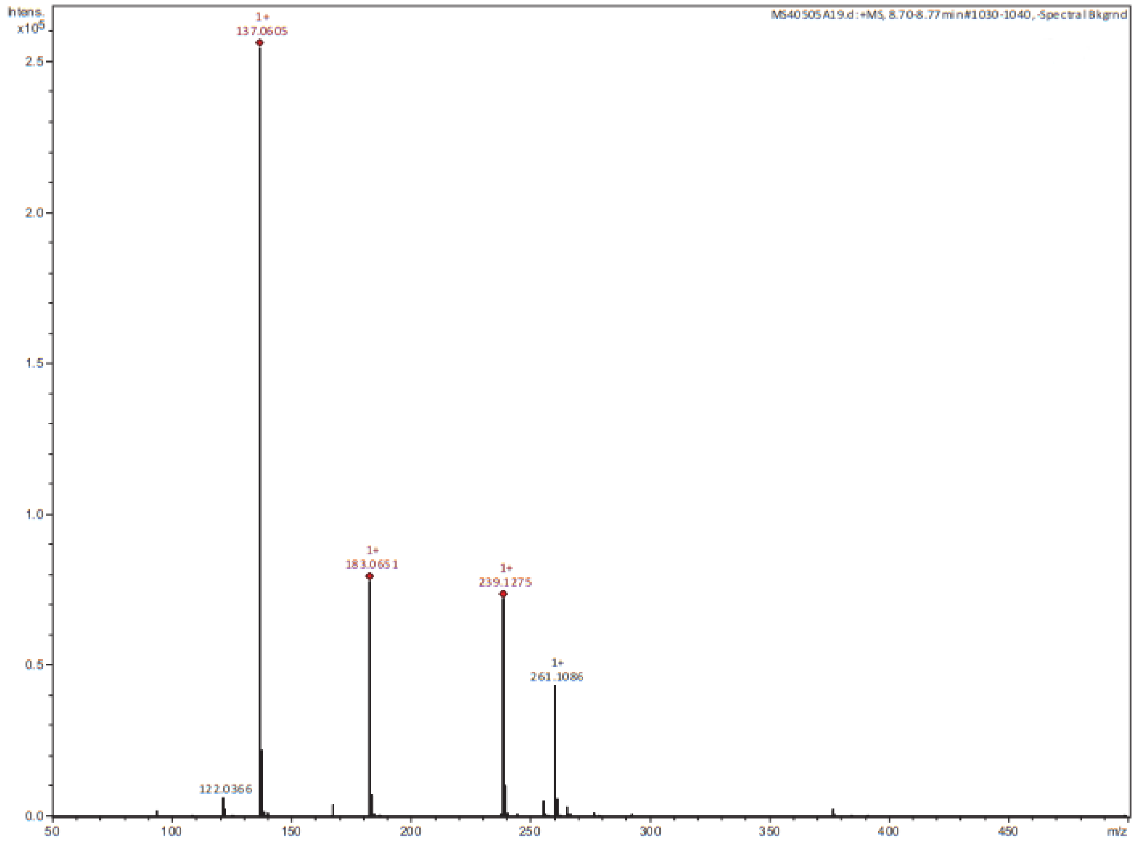

Figure S8: HR-ESIMS Compound 14

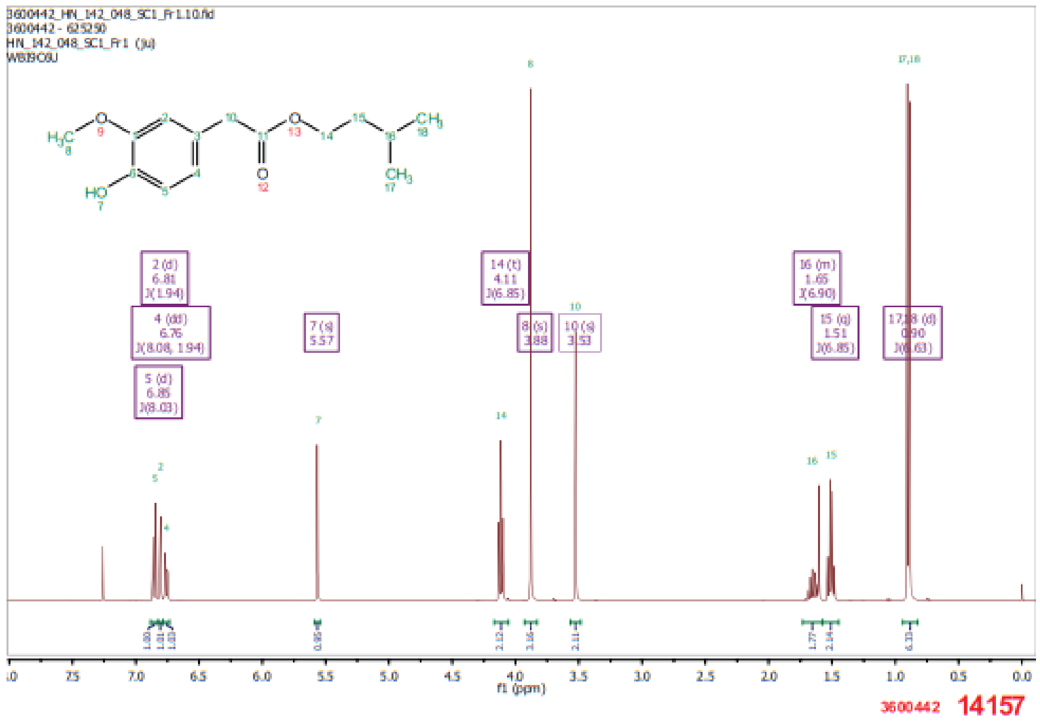

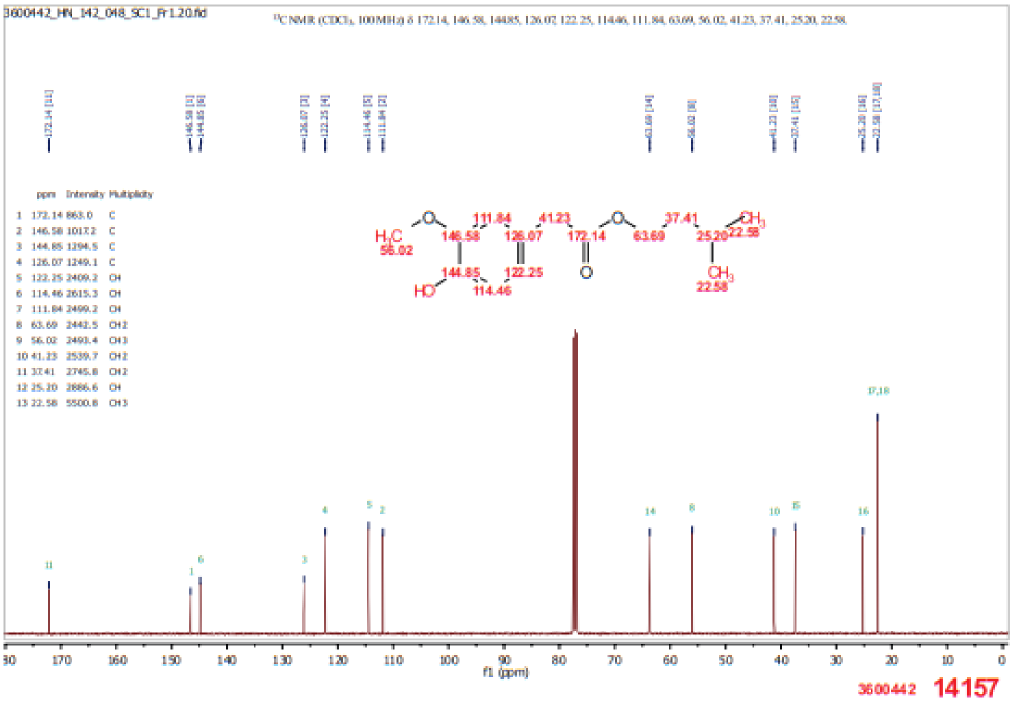

Figure S9: ^{1}H NMR and ^{13}C NMR Compound 15

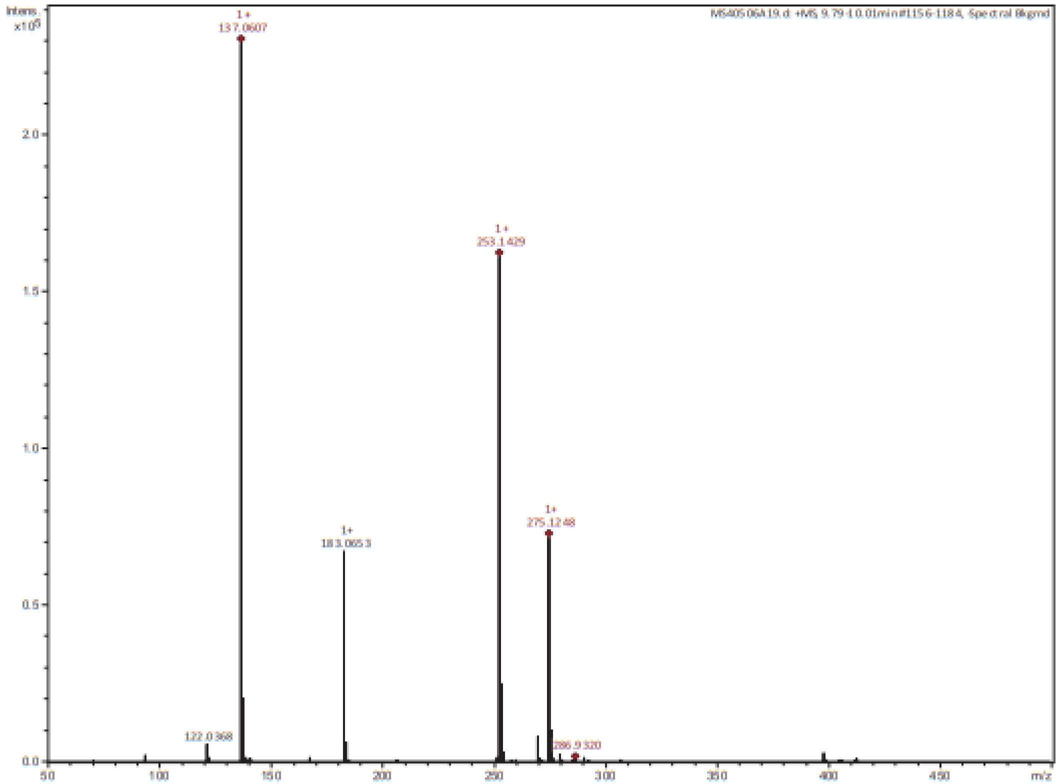

Figure S10: HR-ESIMS Compound 15

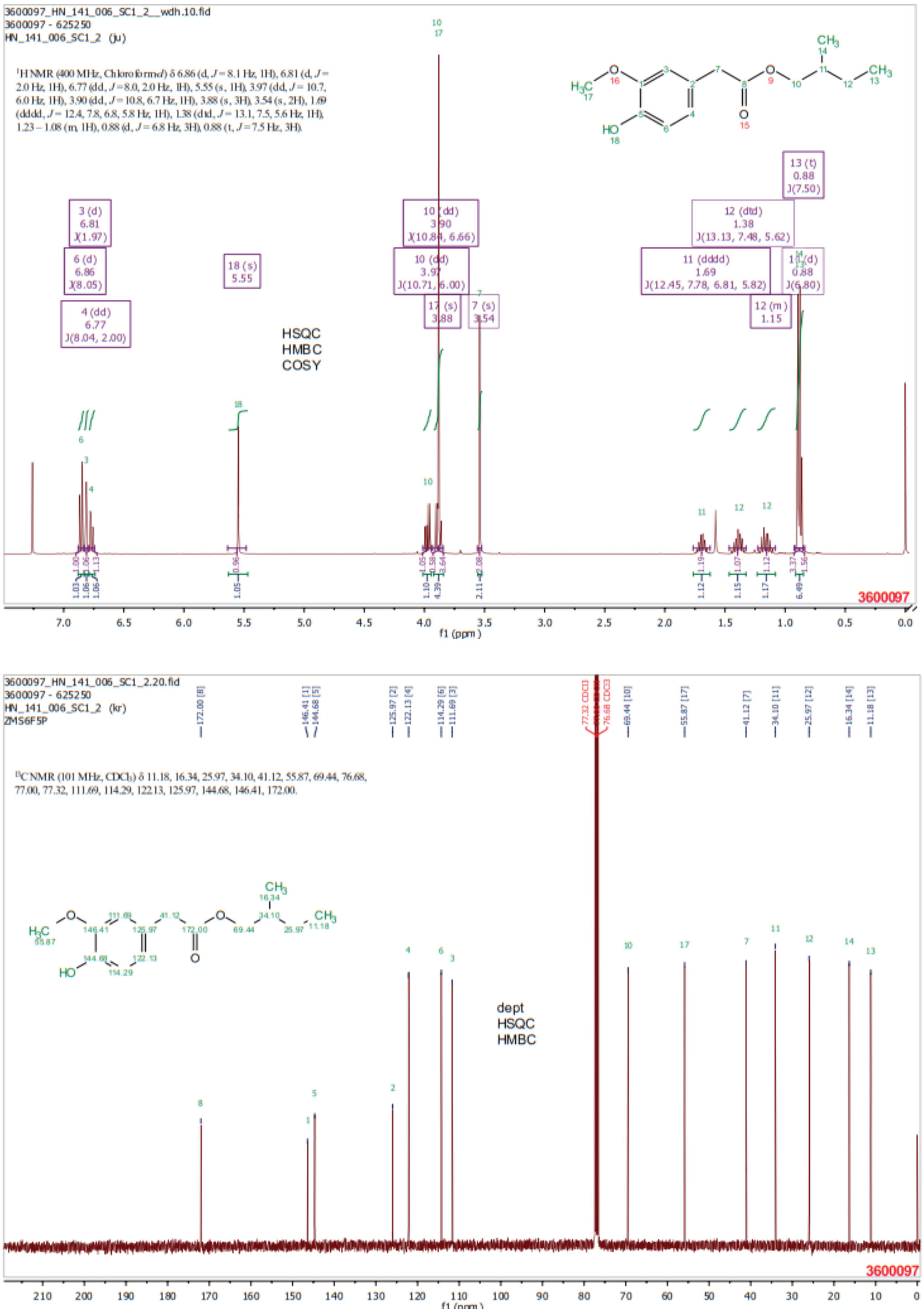

Figure S11: ^{1}H NMR and ^{13}C NMR Compound 16

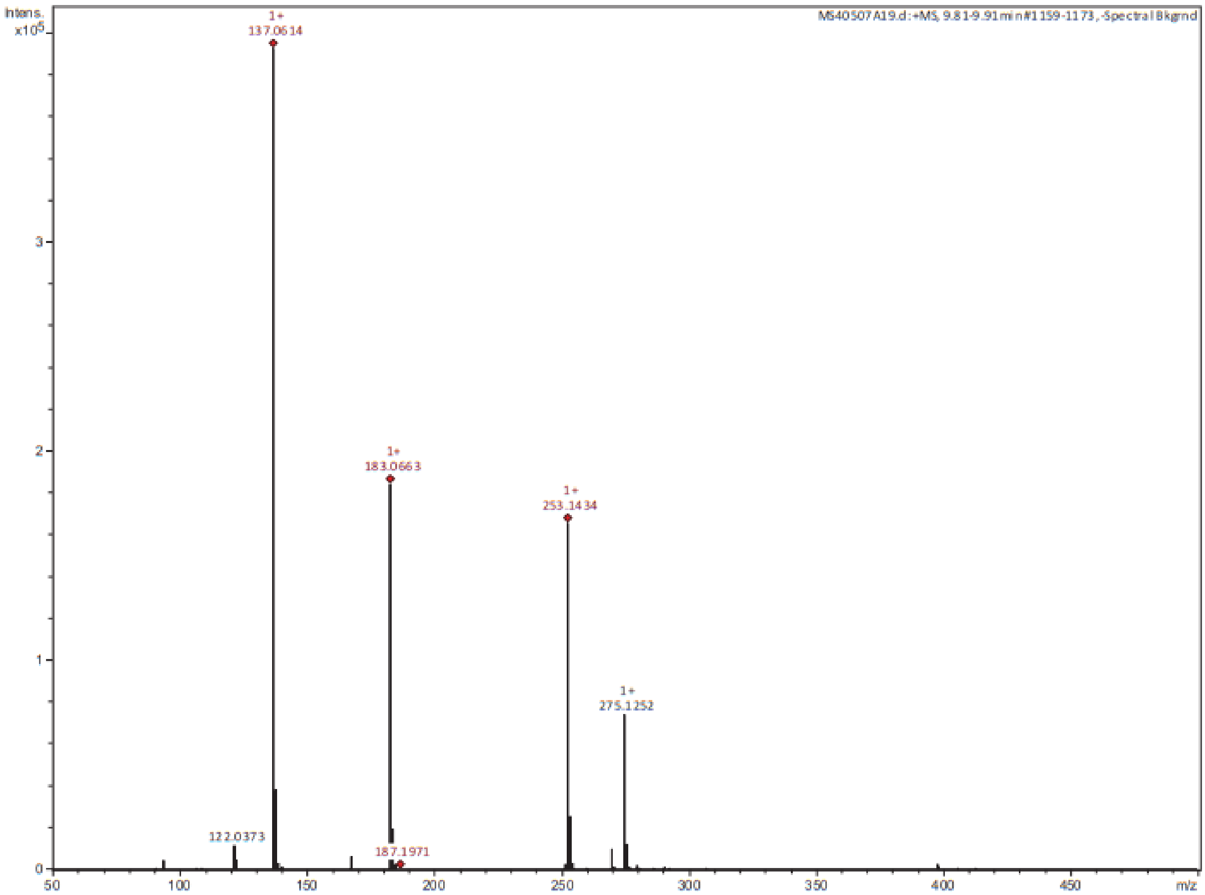

Figure S12: HR-ESIMS Compound 16

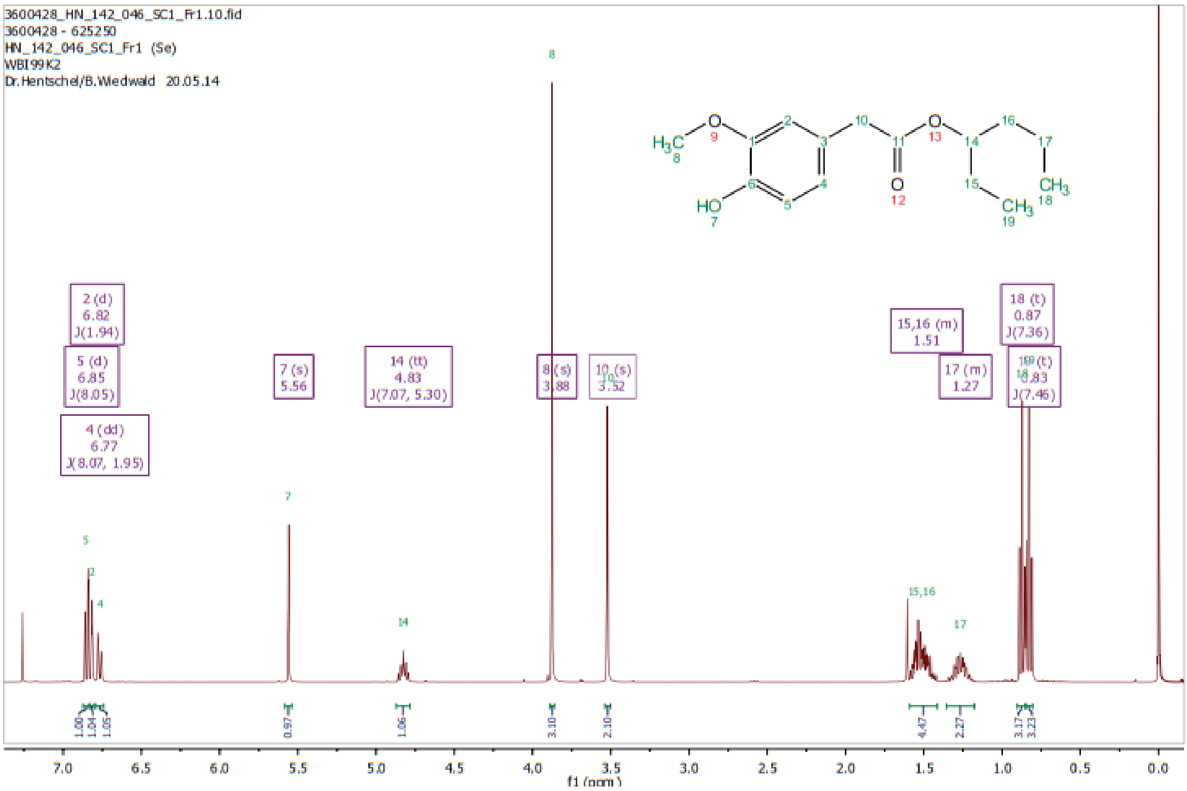

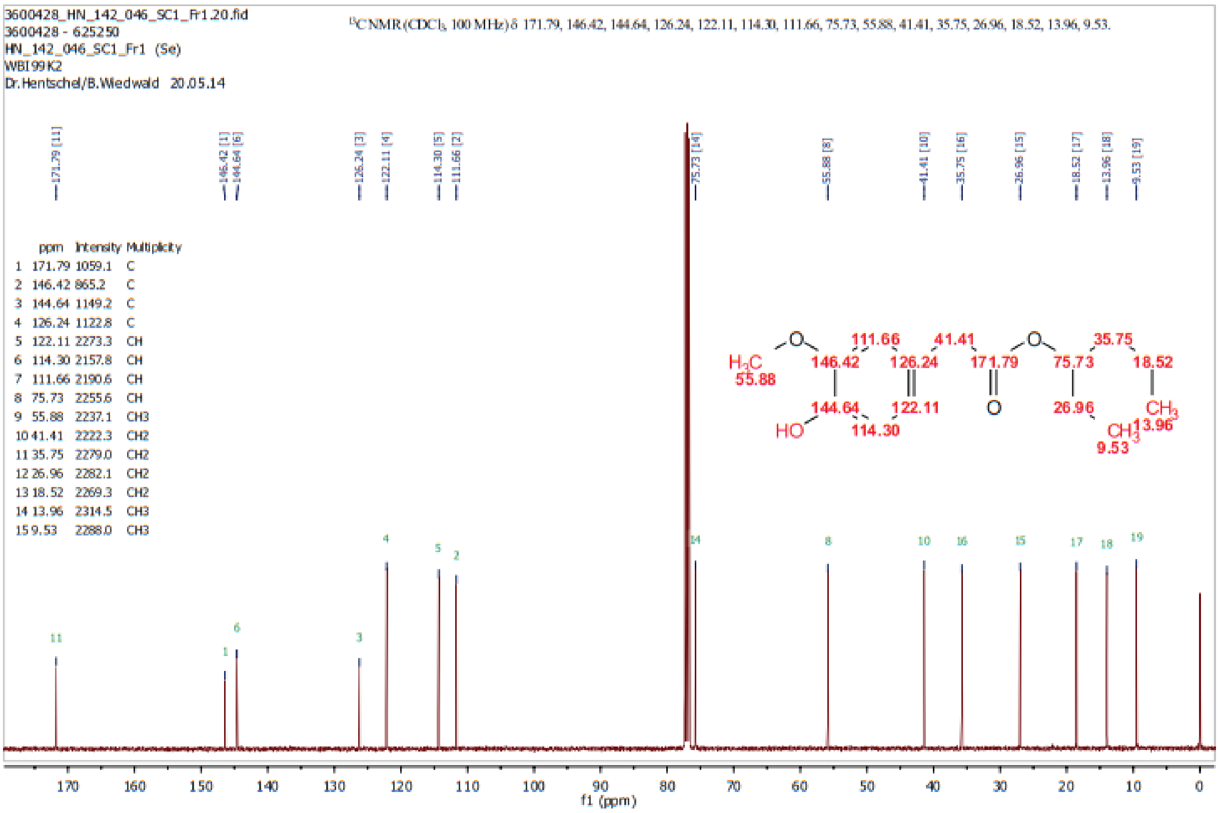

Figure S13: ^{1}H NMR and ^{13}C NMR Compound 17

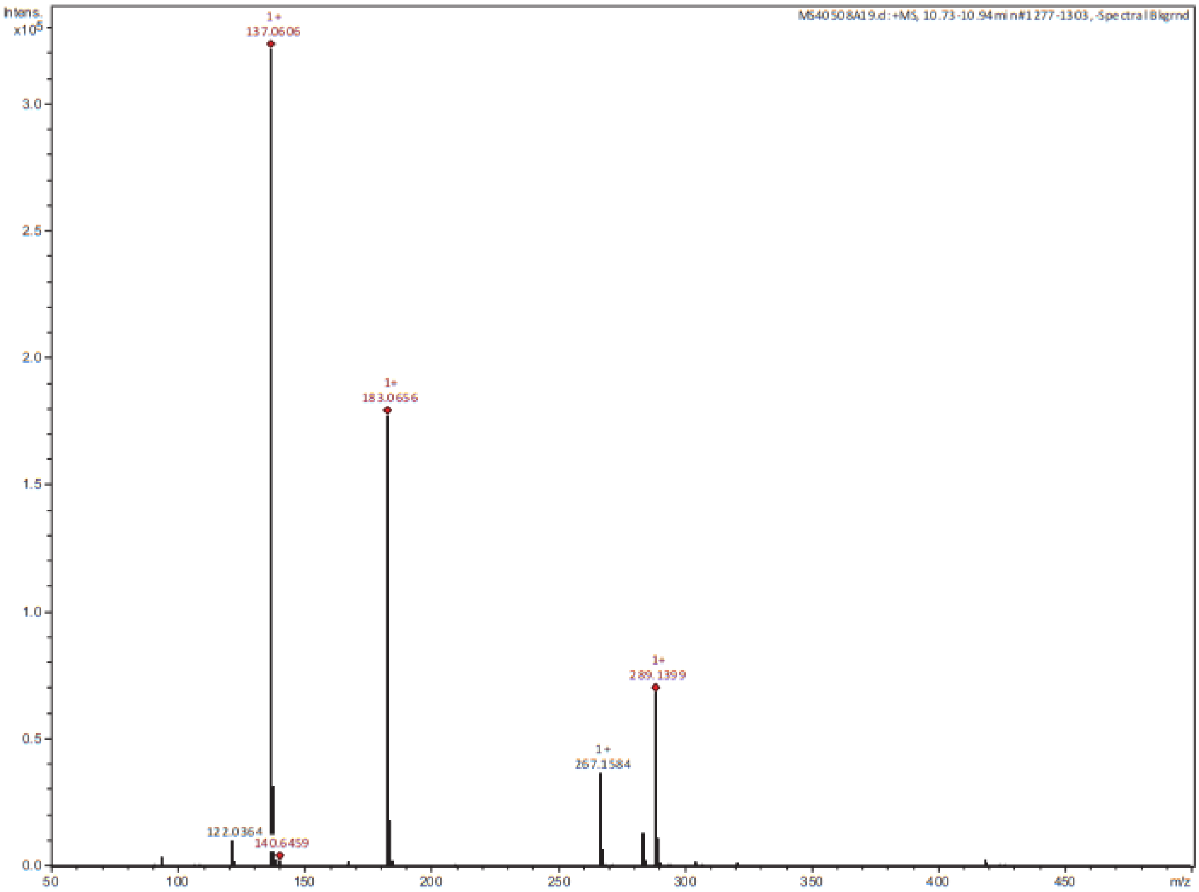

Figure S14: HR-ESIMS Compound 17

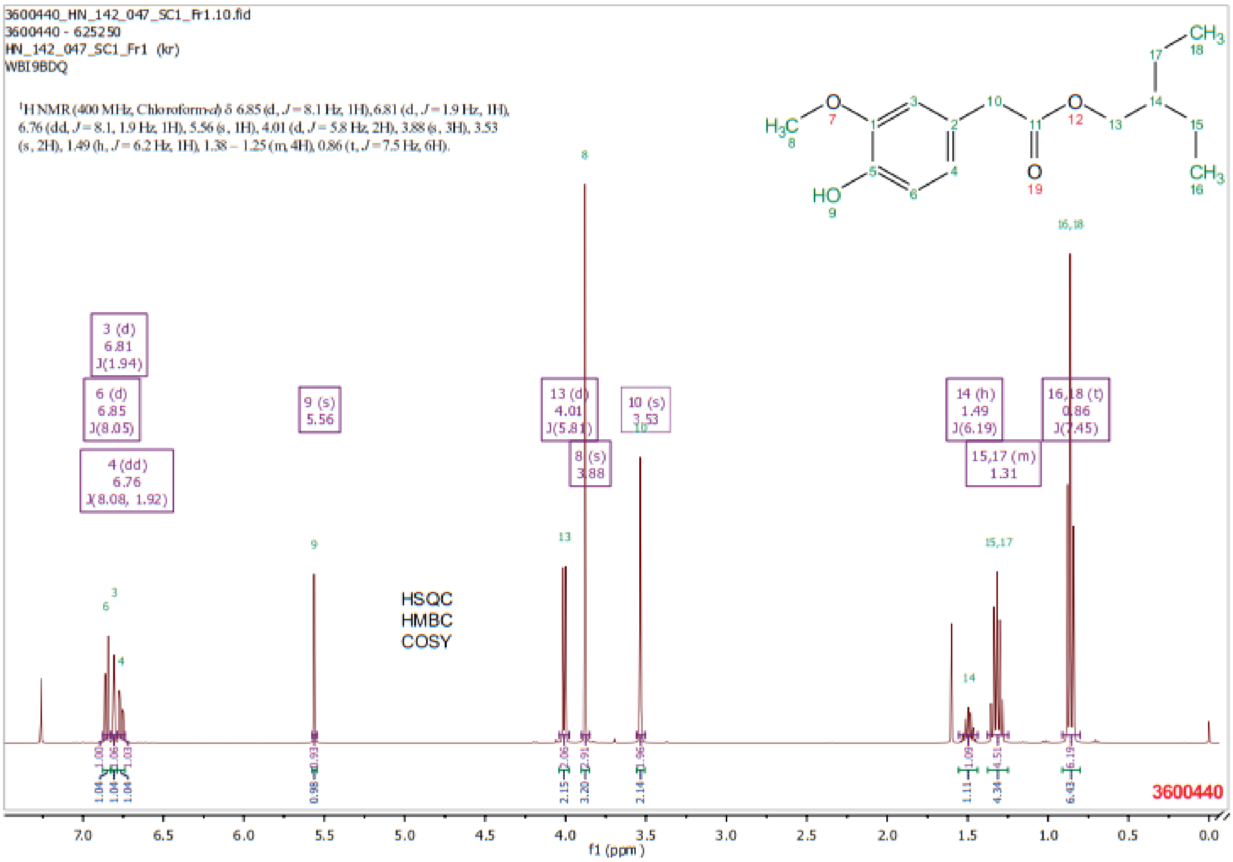

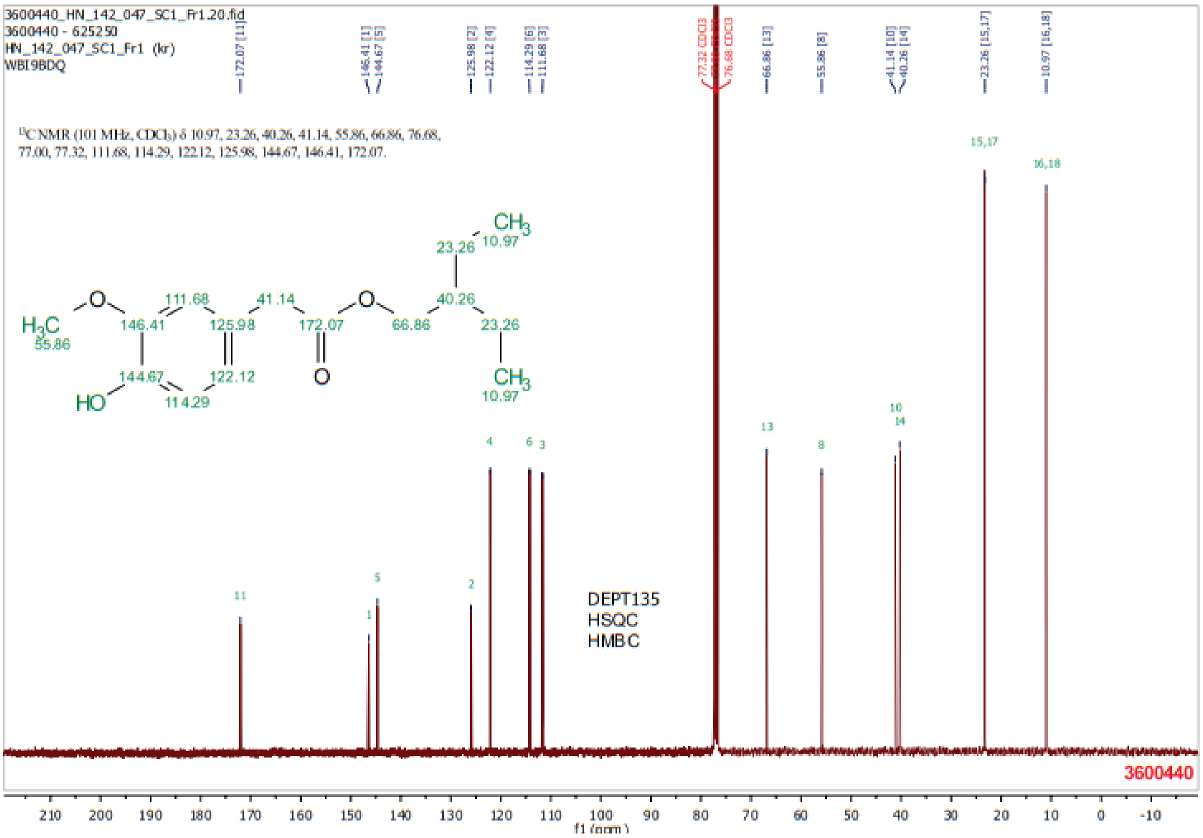

Figure S15: ^{1}H NMR and ^{13}C NMR Compound 18

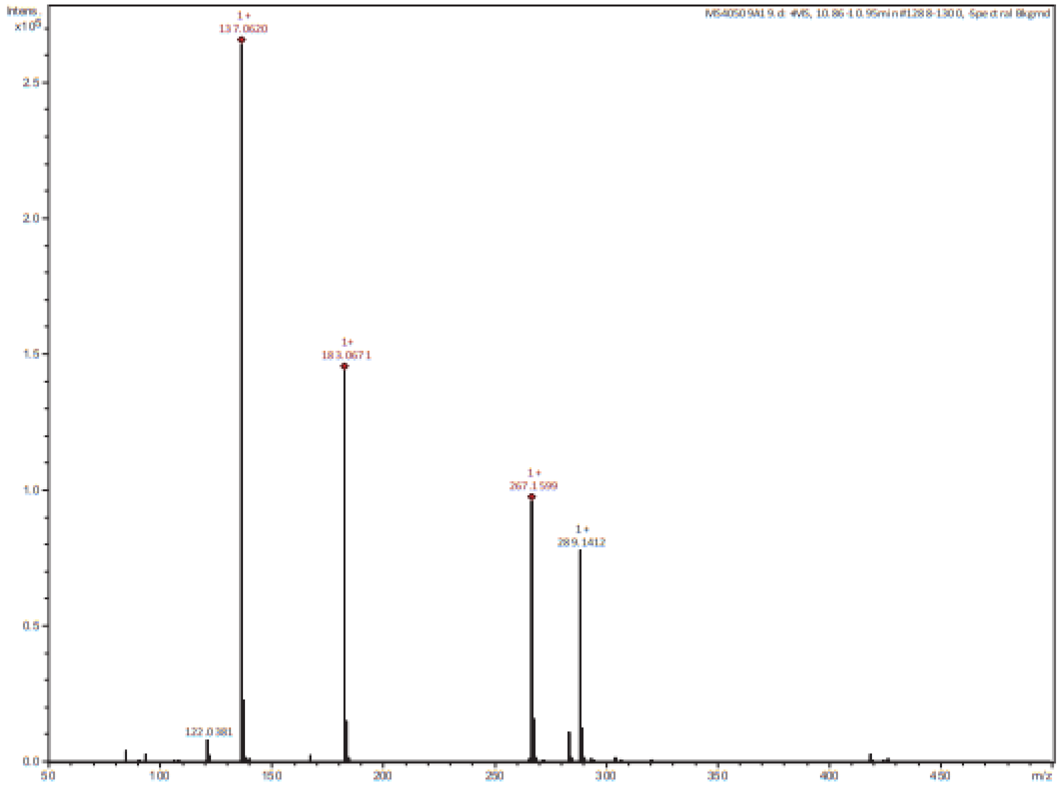

Figure S16: HR-ESIMS Compound 18

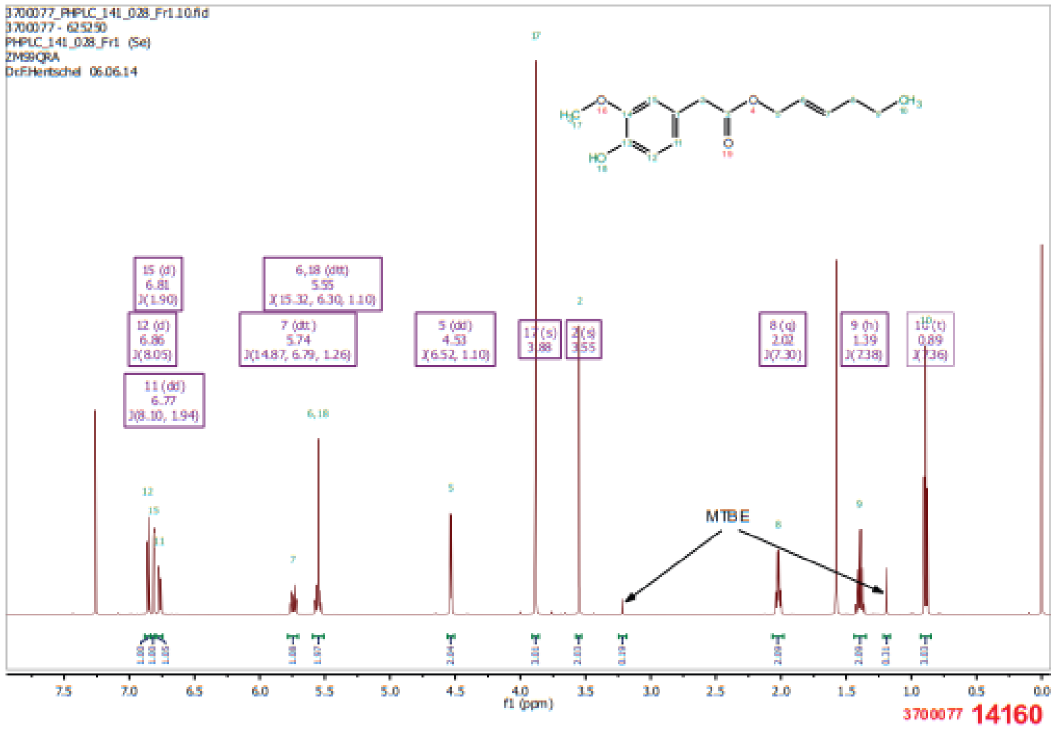

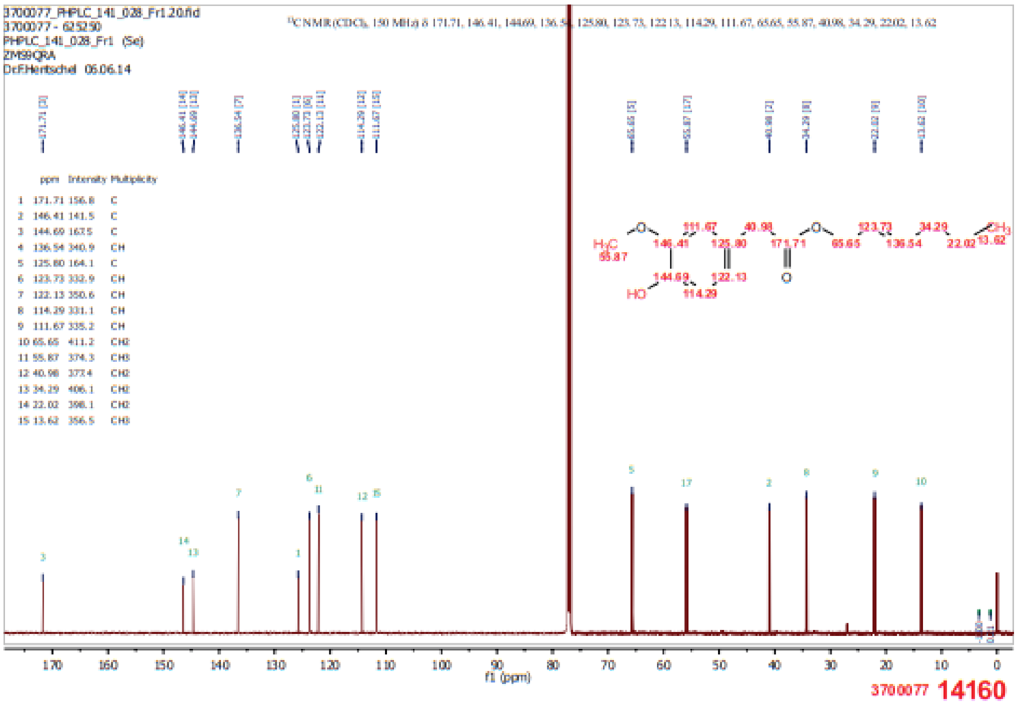

Figure S17: ^{1}H NMR and ^{13}C NMR Compound 19

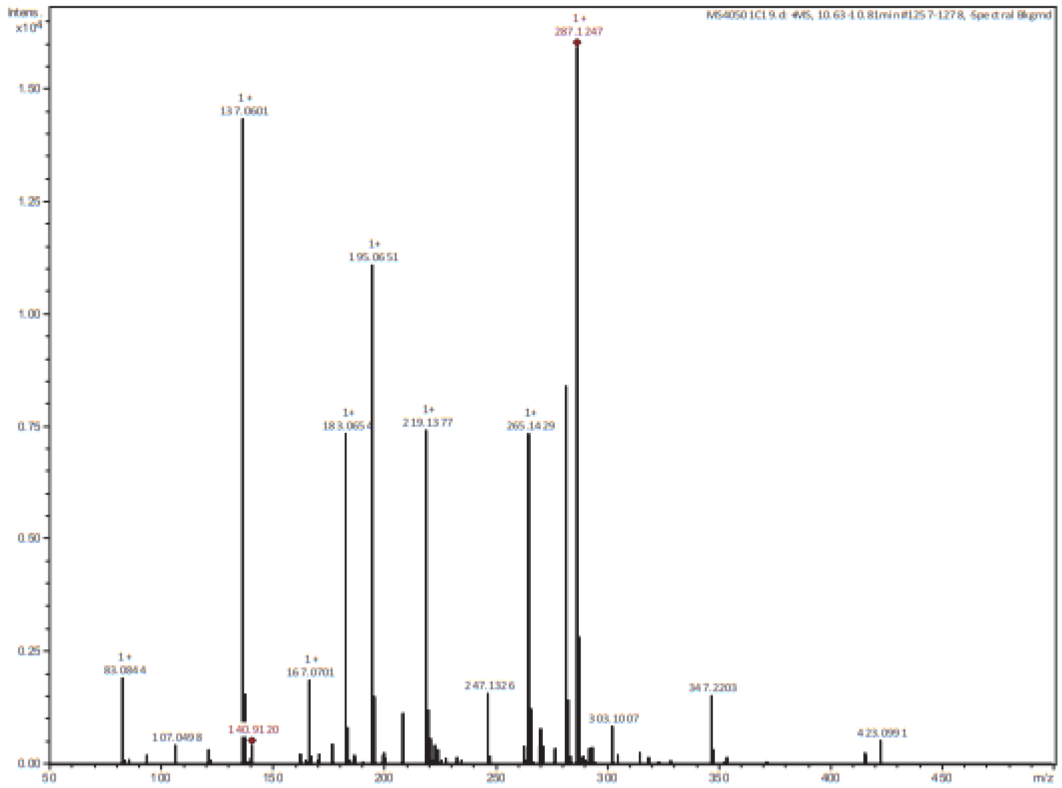

Figure S18: HR-ESIMS Compound 19

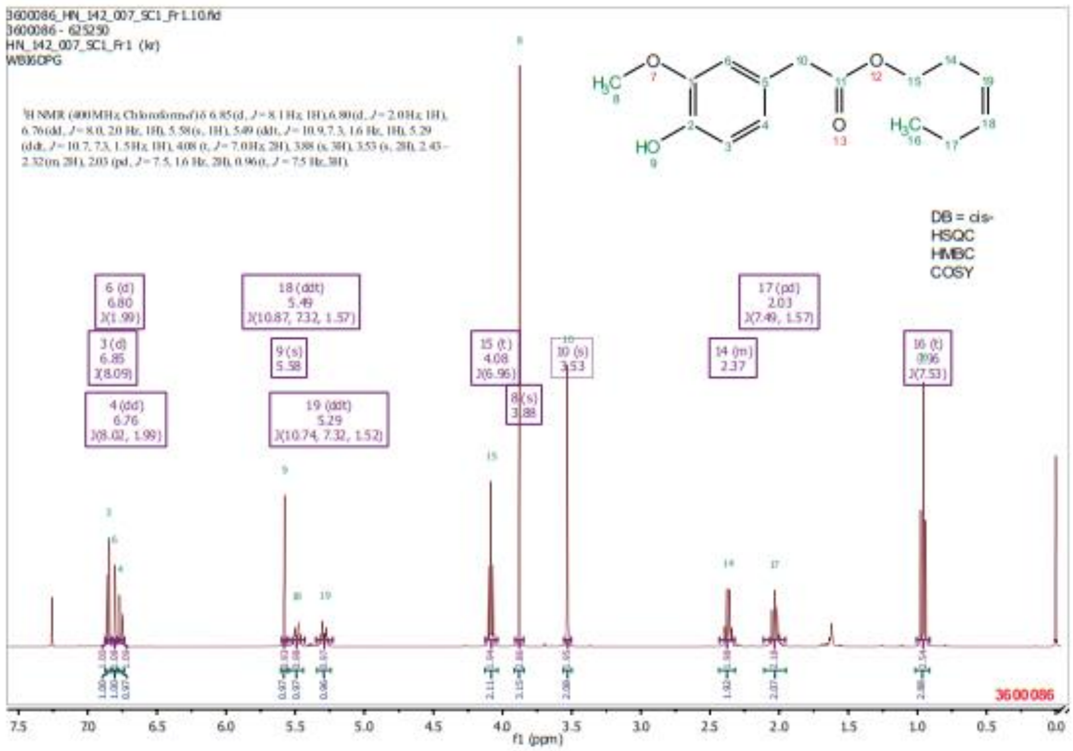

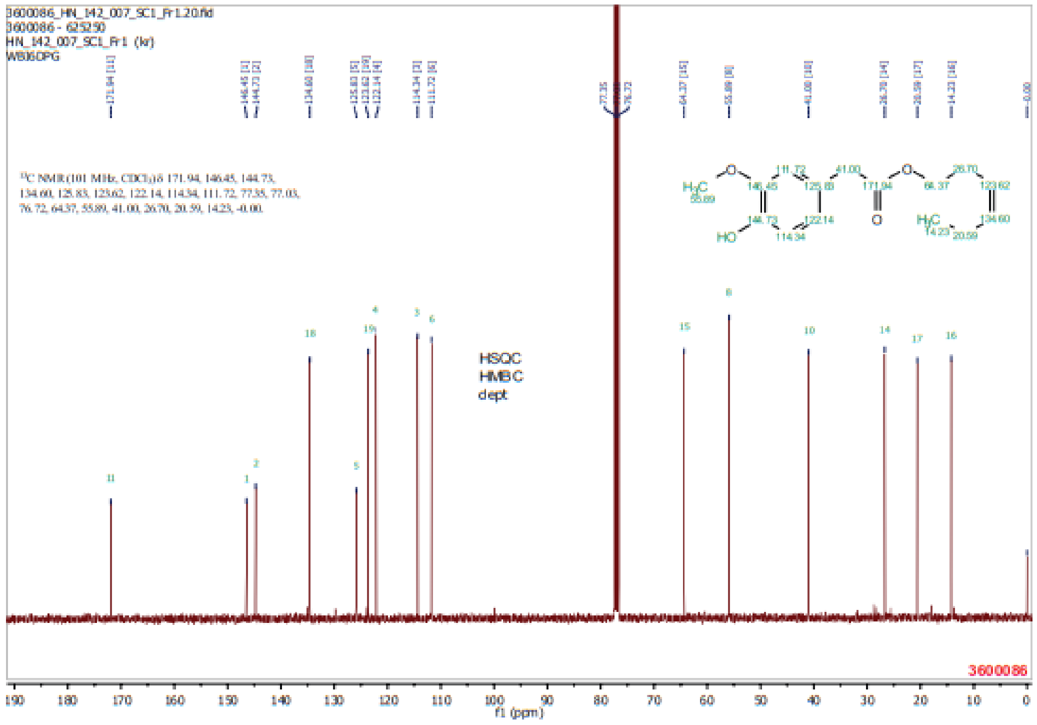

Figure S19: ^{1}H NMR and ^{13}C NMR Compound 20

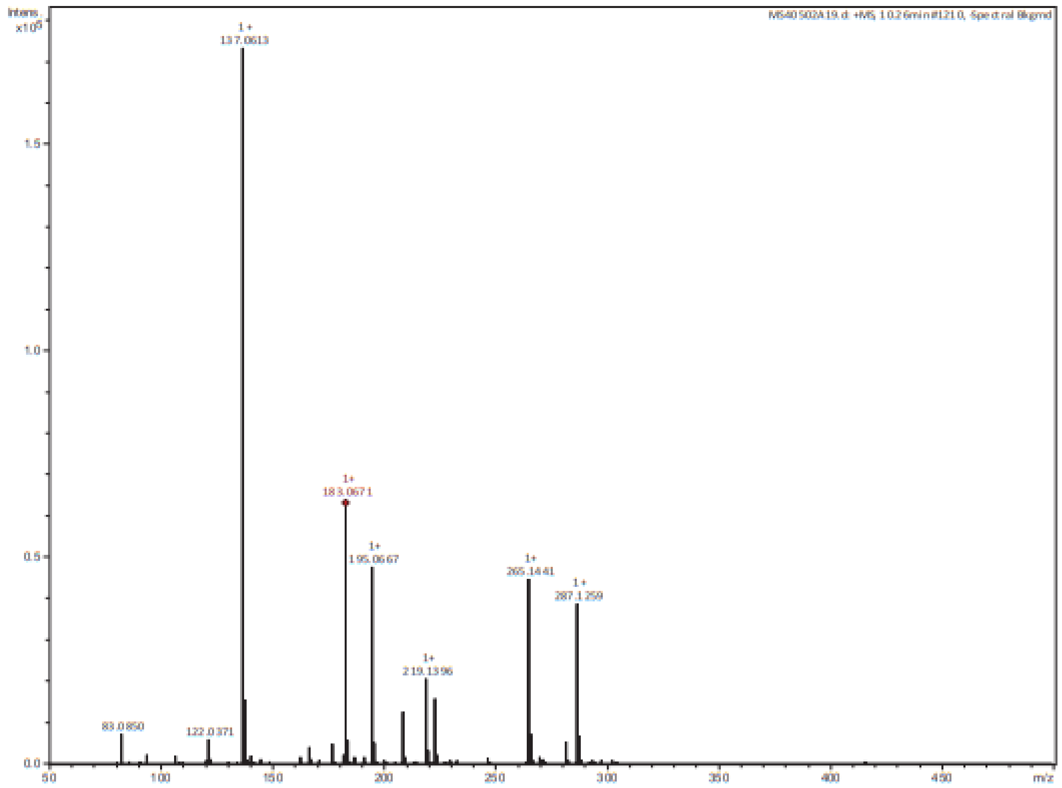

Figure S20: HR-ESIMS Compound 20

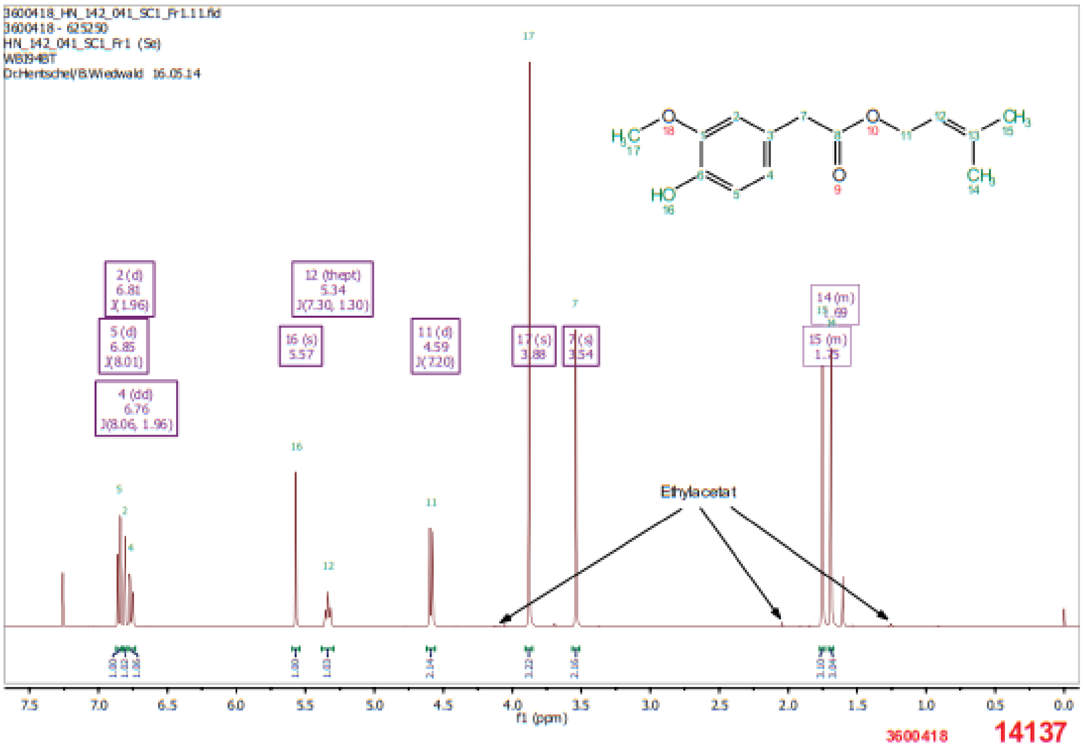

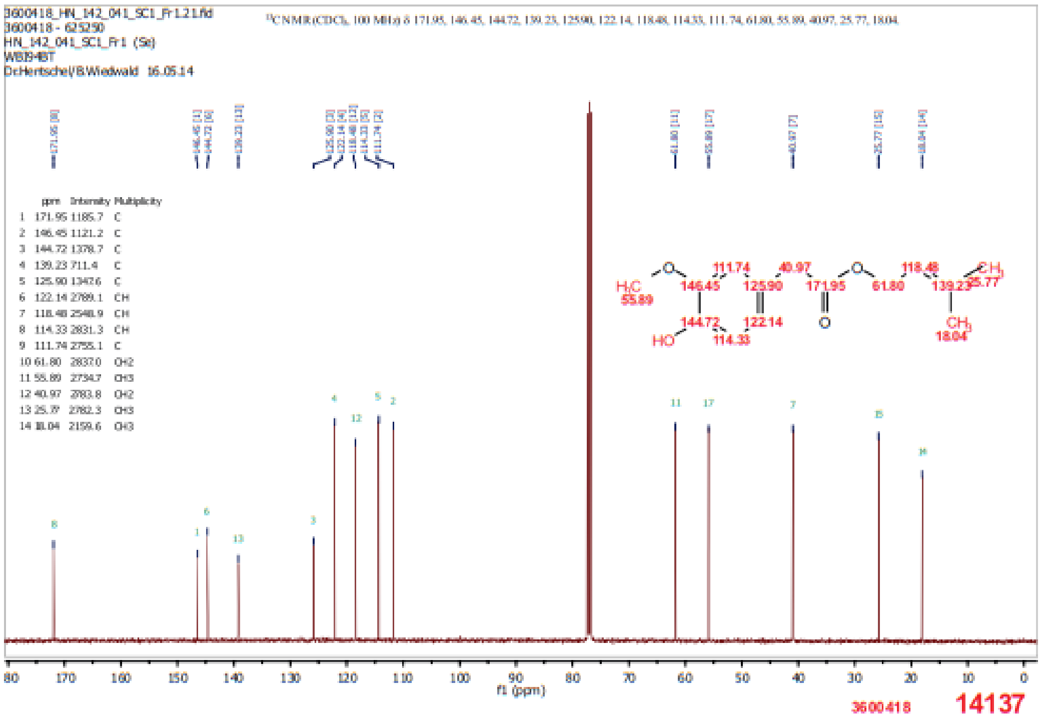

Figure S21: ^{1}H NMR and ^{13}C NMR Compound 21

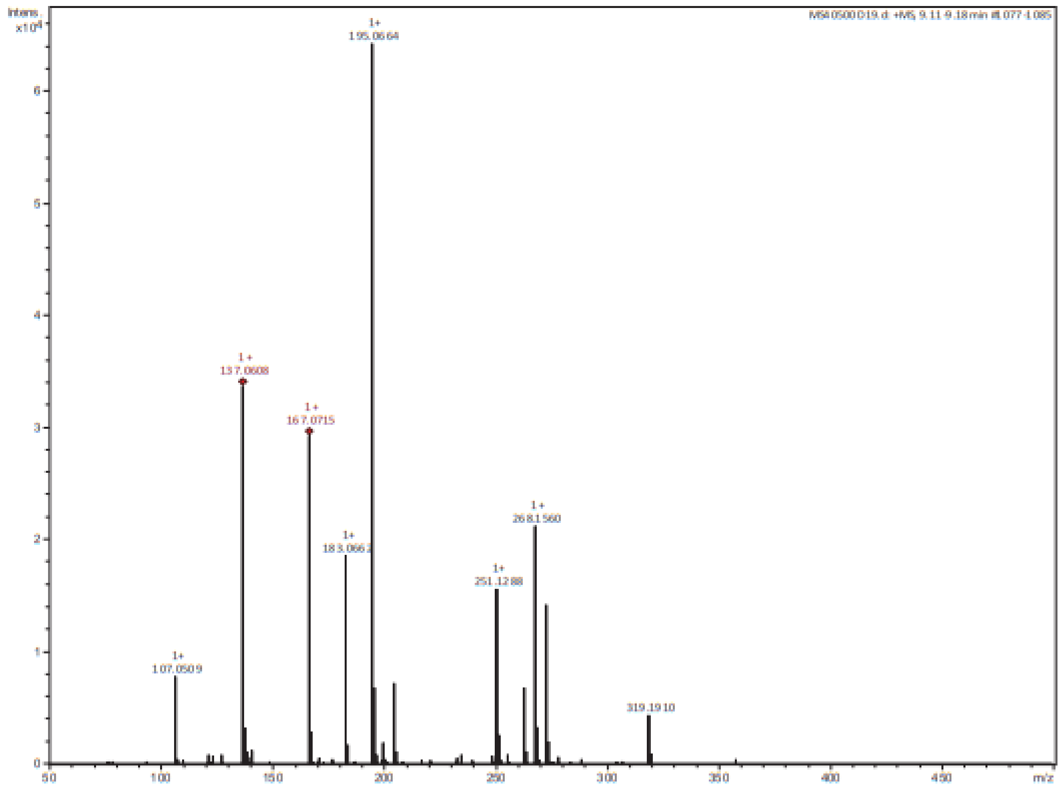

Figure S22: HR-ESIMS Compound 21

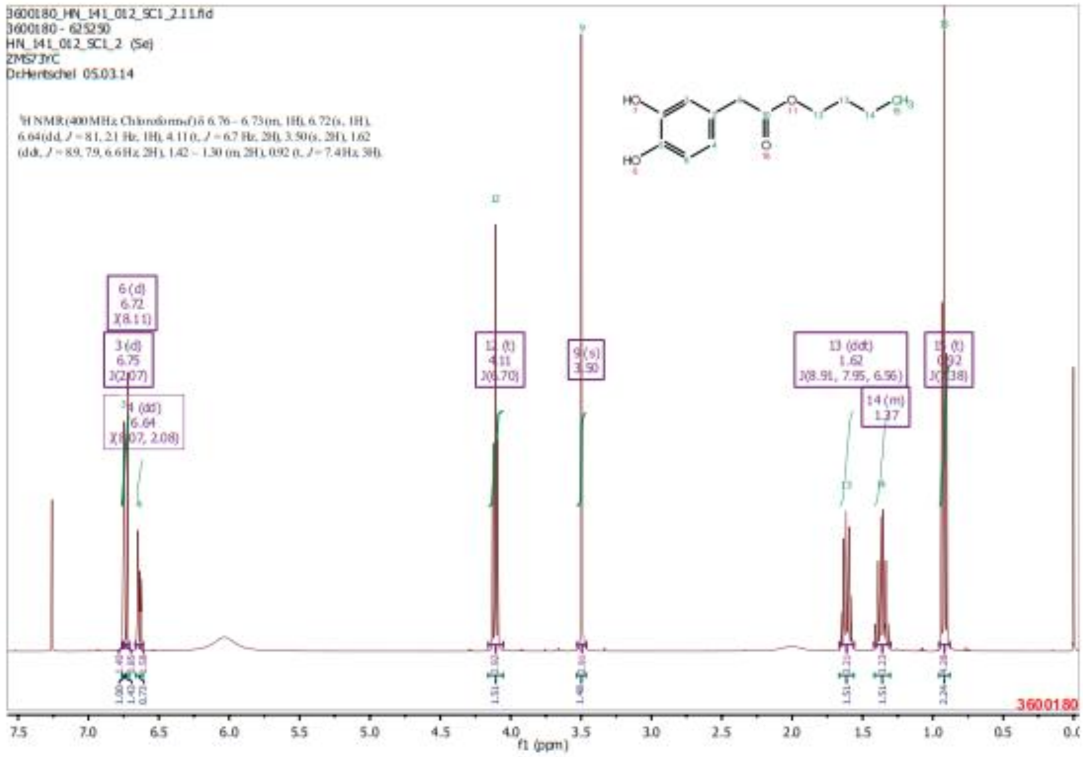

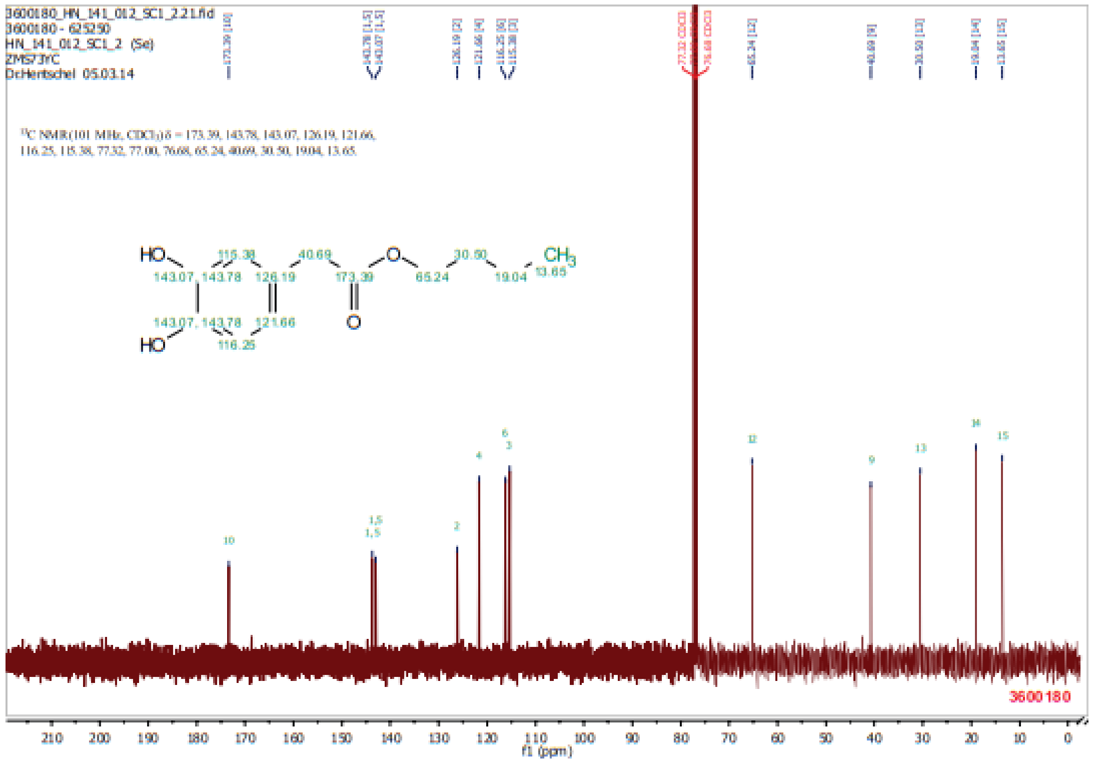

Figure S23: ^{1}H NMR and ^{13}C NMR Compound 23

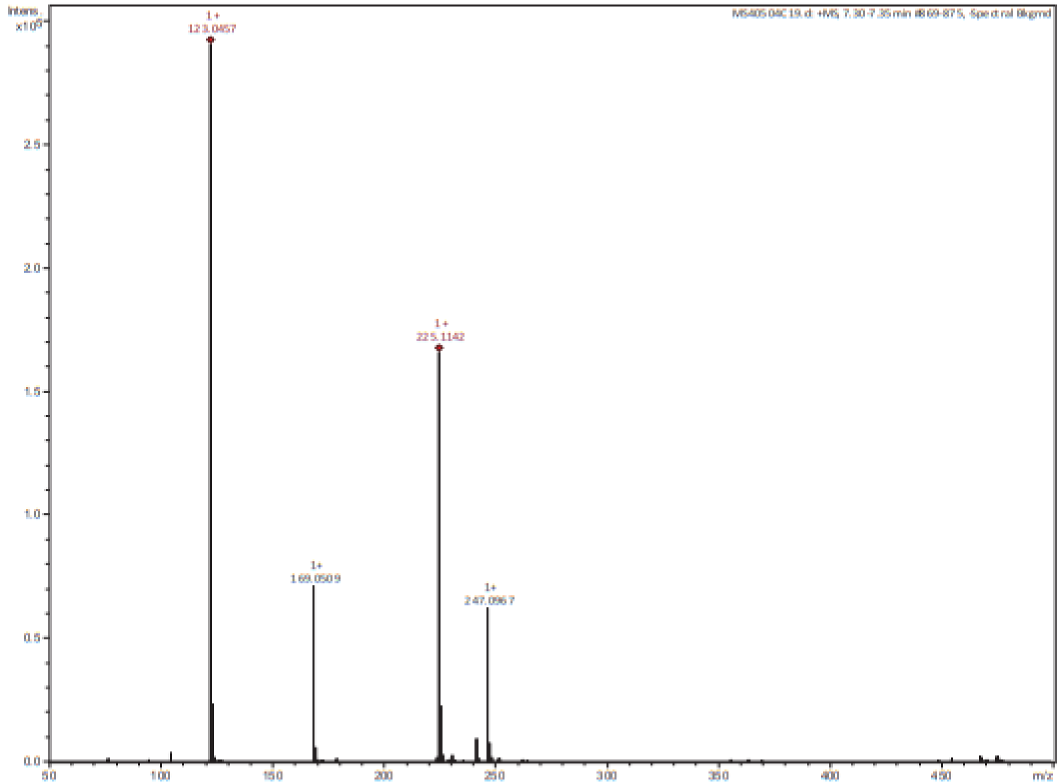

Figure S24: HR-ESIMS Compound 23
